# Supplementary material for: AlphaFold-SFA: Accelerated sampling of cryptic pocket opening, protein-ligand binding and allostery by AlphaFold, slow feature analysis and metadynamics
Source: PLoS One. 2024 Aug 27;19(8):e0307226. doi: 10.1371/journal.pone.0307226 (PMC11349229; doi:10.1371/journal.pone.0307226)
Supplement: S1 Fig — (A)Markov state model was generated using AlphaFold seeded unbiased molecular dynamics simulations (80 structures * 2 independent clones * 100ns each = total 16 μs) reported by Meller and coworkers. MSM was performed on sin and cos transformed χ1 and χ2 angles of Trp41. (B) The convergence of MSM has been highlighted by capturing timescale associated with conformational transition with different choices of clusters (k). Timescales associated with conformational transitions between open and closed states are in the same order of magnitude highlighting the convergence of MSM. (PDF) [file pone.0307226.s001.pdf]

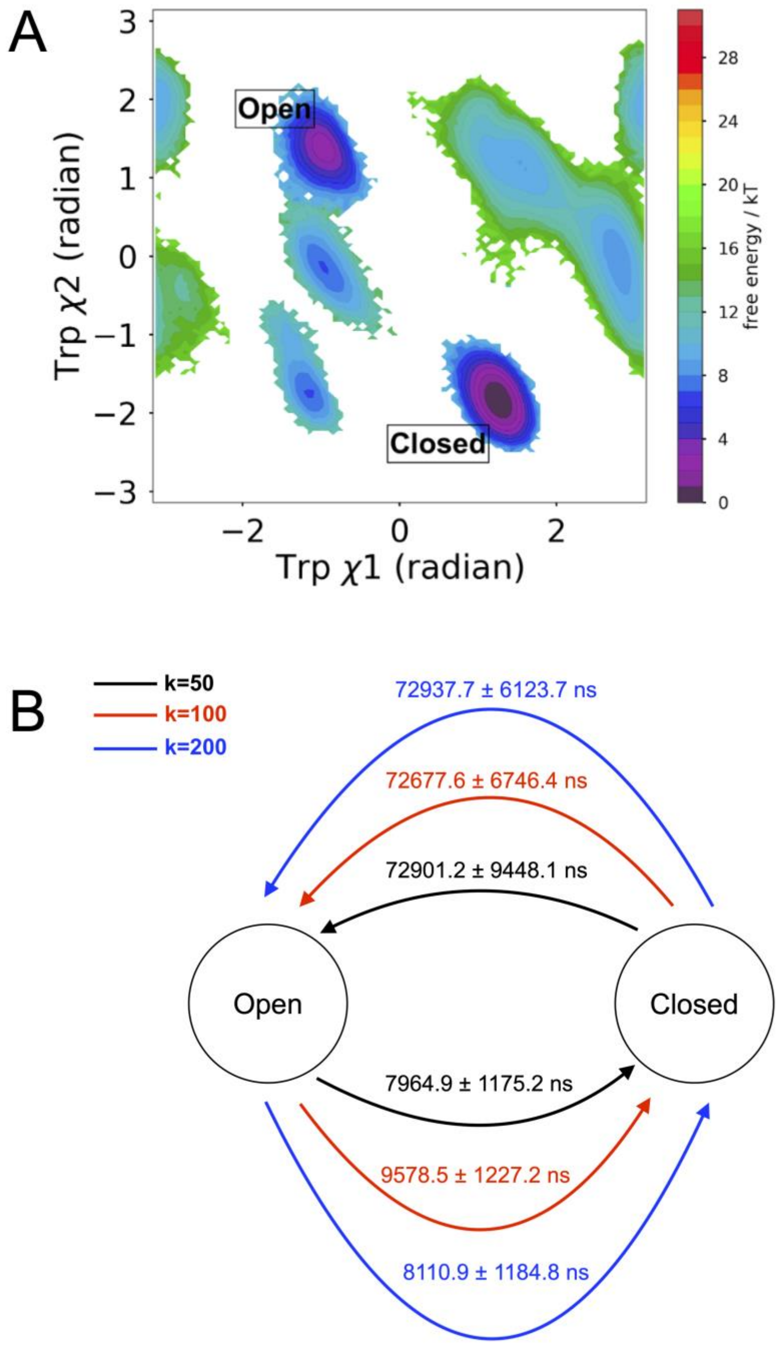

S1 Fig. **MSM weighted equilibrium population projected along Trp41 $\chi 1$ and $\chi 2$ highlighted sampling of closed and open states of plasmepsin II**.

(A)Markov state model was generated using AlphaFold seeded unbiased molecular dynamics simulations (80 structures * 2 independent clones * 100ns each = total 16 μs) reported by *Meller and coworkers*. MSM was performed on sin and cos transformed $\chi 1$ and $\chi 2$ angles of Trp41. (B) The convergence of MSM has been highlighted by capturing timescale associated with conformational transition with different choices of clusters (k). Timescales associated with conformational transitions between *open* and *closed* states are in the same order of magnitude highlighting the convergence of MSM.
