# Supplementary material for: AlphaFold-SFA: Accelerated sampling of cryptic pocket opening, protein-ligand binding and allostery by AlphaFold, slow feature analysis and metadynamics
Source: PLoS One. 2024 Aug 27;19(8):e0307226. doi: 10.1371/journal.pone.0307226 (PMC11349229; doi:10.1371/journal.pone.0307226)
Supplement: S2 Fig — (A) Implied timescale plot associated with Markov state model. We chose lag time of 6ns to generate MSM. (B) PCCA+ was used to generate microstate definition associated with Trp41 χ1 and χ2 angles. PCCA+ manage to separate closed (S6) and open (S5) states in plasmepsin II. (PDF) [file pone.0307226.s002.pdf]

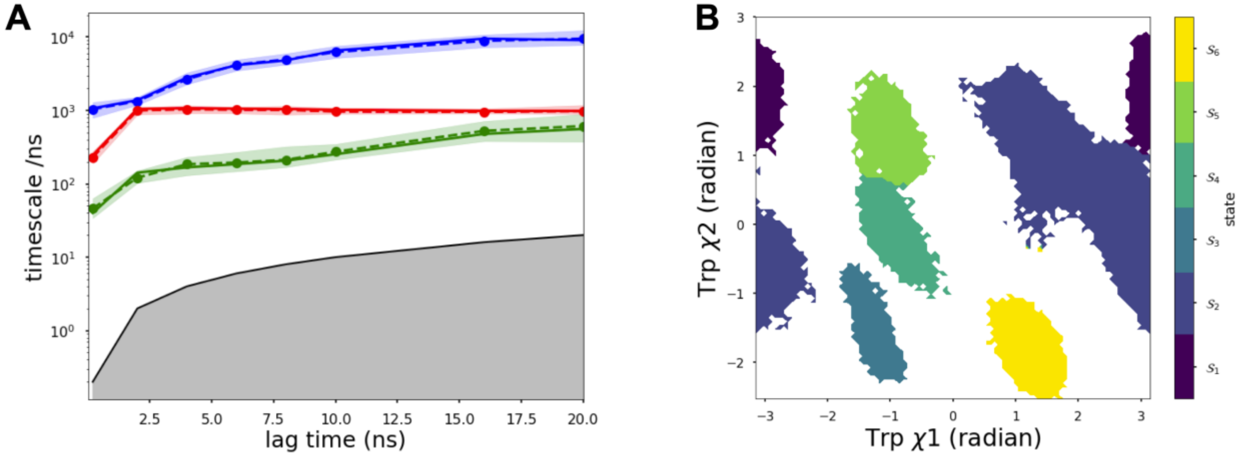

S2 Fig. Implied timescales associated with the MSM

(A) Implied timescale plot associated with Markov state model. We chose lag time of 6ns to generate MSM. (B) *$PCCA^+$* was used to generate microstate definition associated with Trp41 $\chi 1$ and $\chi 2$ angles. *$PCCA^+$* manage to separate closed (*S6*) and open (*S5*) states in plasmepsin II.
