# Supplementary material for: AlphaFold-SFA: Accelerated sampling of cryptic pocket opening, protein-ligand binding and allostery by AlphaFold, slow feature analysis and metadynamics
Source: PLoS One. 2024 Aug 27;19(8):e0307226. doi: 10.1371/journal.pone.0307226 (PMC11349229; doi:10.1371/journal.pone.0307226)
Supplement: S3 Fig — (A) SF1 Weights corresponding each feature. (B) SF2 weights corresponding each feature. List of features can be accessed here: https://github.com/sbhakat/AlphaFold-SFA/blob/main/Plm-cryptic-pocket/features.ipynb It highlights how first two slow features (A, B) manage to capture sidechain flipping associated with Trp41 and Tyr77, χ1 and χ2 angles. (PDF) [file pone.0307226.s003.pdf]

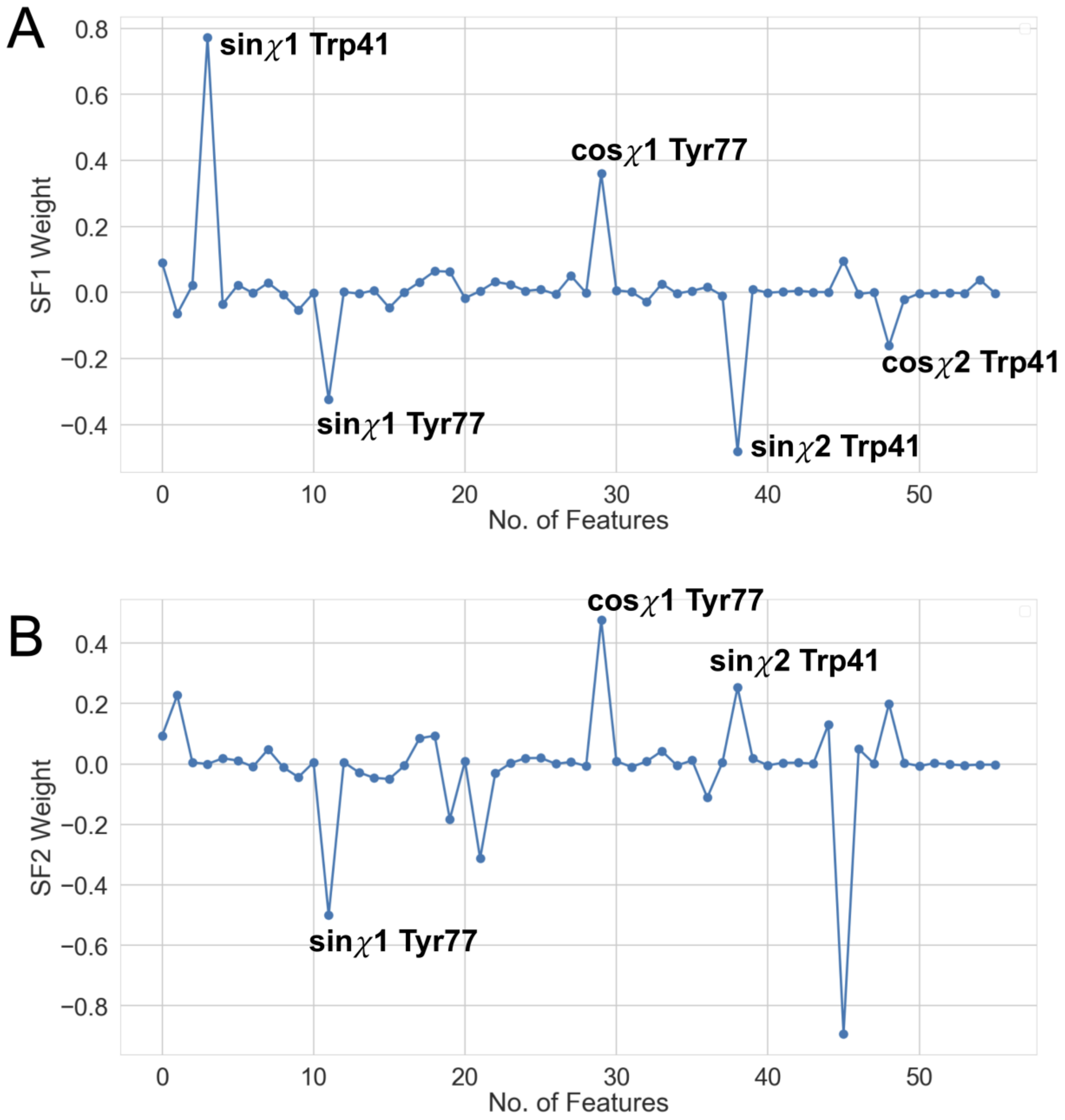

S3 Fig. SFA weights and corresponding features.

(A) SF1 Weights corresponding each feature. (B) SF2 weights corresponding each feature. List of features can be accessed here: https://github.com/sbhakat/AlphaFold-SFA/blob/main/Plm-cryptic-pocket/features.ipynb It highlights how first two slow features (A, B) manage to capture sidechain flipping associated with Trp41 and Tyr77, $\chi1$ and $\chi2$ angles.
