# Supplementary material for: AlphaFold-SFA: Accelerated sampling of cryptic pocket opening, protein-ligand binding and allostery by AlphaFold, slow feature analysis and metadynamics
Source: PLoS One. 2024 Aug 27;19(8):e0307226. doi: 10.1371/journal.pone.0307226 (PMC11349229; doi:10.1371/journal.pone.0307226)
Supplement: S4 Fig — (A) Projection of first two slow features along sin transformed χ1 angle of Trp41 highlights from the training data highlights separation along Trp41, a key residue involved in cryptic pocket opening. (B) Projection of Trp41 χ1 and χ2 angle along SF1. (C) Free energy surface projected on first two slow features (SF1 and SF2) from unbiased MD simulations starting from closed conformation of plasmepsin II. (D) Reweighted free energy surface projected along first two slow features from metadynamics simulations with slow features as CVs highlights accelerated sampling compared to unbiased MD simulations. (PDF) [file pone.0307226.s004.pdf]

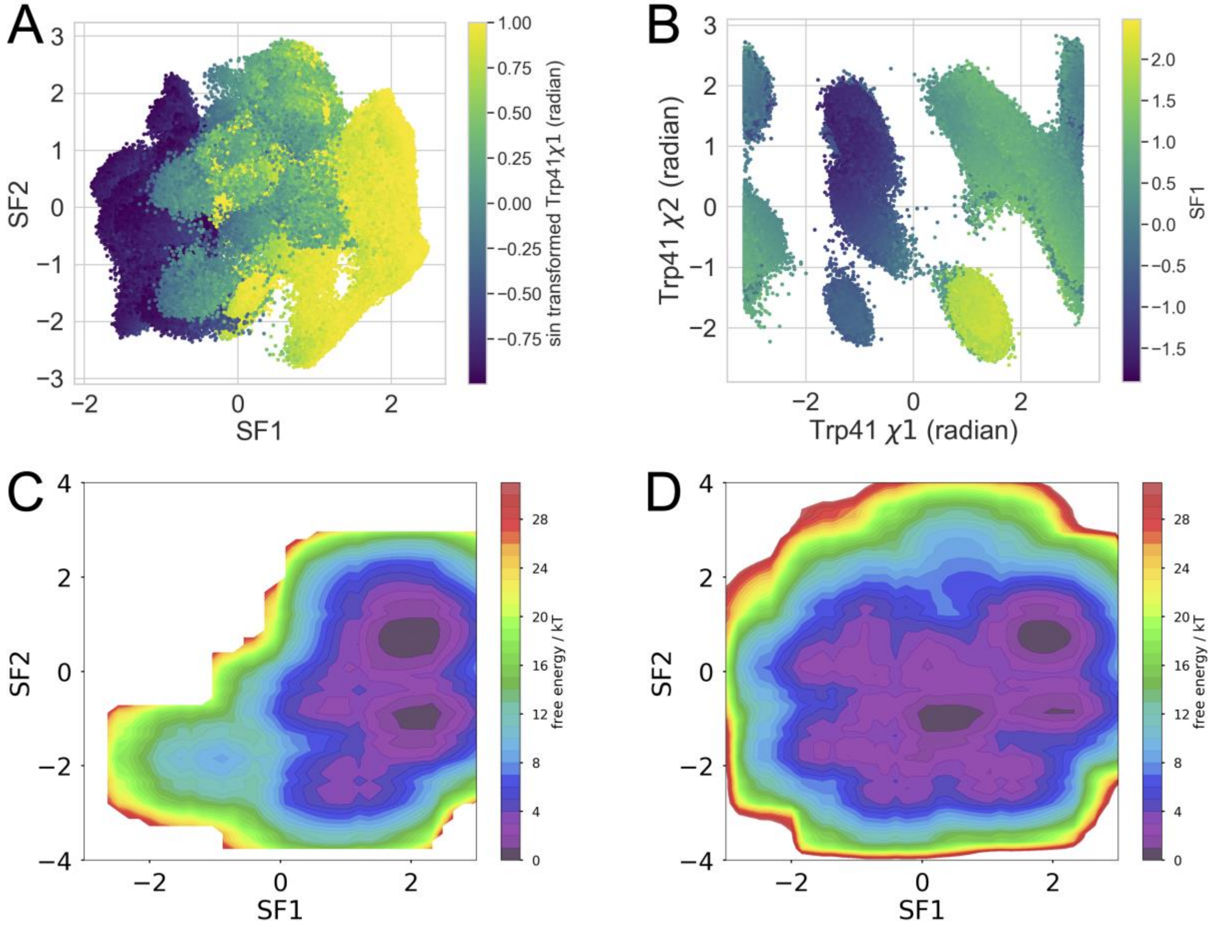

S4 Fig. Projection of SF1 and SF2.

(A) Projection of first two slow features along sin transformed $\chi1$ angle of Trp41 highlights from the training data highlights separation along Trp41, a key residue involved in cryptic pocket opening. (B) Projection of Trp41 $\chi1$ and $\chi2$ angle along SF1. (C) Free energy surface projected on first two slow features (SF1 and SF2) from unbiased MD simulations starting from closed conformation of plasmepsin II. (D) Reweighted free energy surface projected along first two slow features from metadynamics simulations with slow features as CVs highlights accelerated sampling compared to unbiased MD simulations.
