# Supplementary material for: AlphaFold-SFA: Accelerated sampling of cryptic pocket opening, protein-ligand binding and allostery by AlphaFold, slow feature analysis and metadynamics
Source: PLoS One. 2024 Aug 27;19(8):e0307226. doi: 10.1371/journal.pone.0307226 (PMC11349229; doi:10.1371/journal.pone.0307226)
Supplement: S5 Fig — (A) Time trace SF1 in the training data. (B) Time traced of SF1 in SFA-metadynamics. (C) Time trace of SF2 in the training data. (D) Time trace of SF2 in SFA-metadynamics. Metadynamics simulations using slow features as CVs manage to capture multiple recrossing within a few hundreds of nanoseconds (C, D). (PDF) [file pone.0307226.s005.pdf]

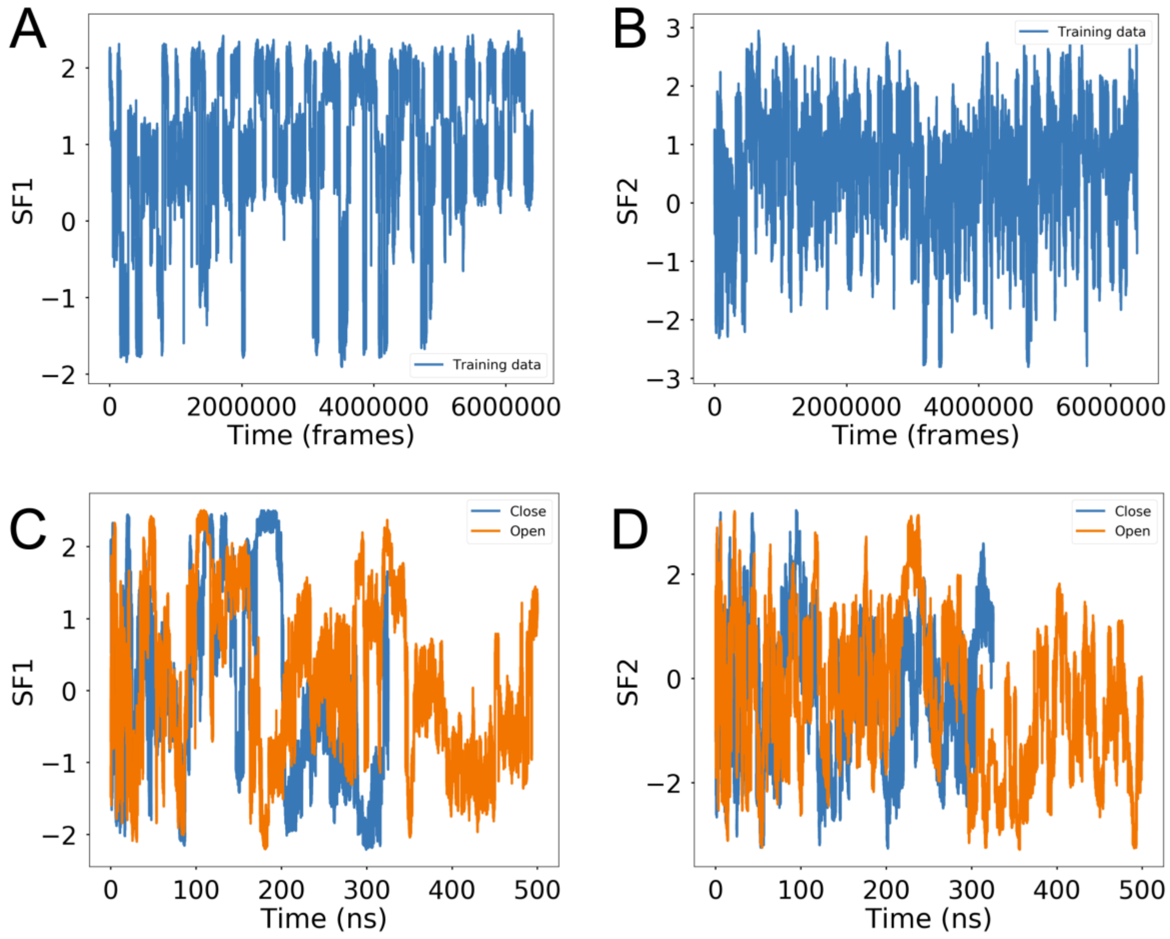

S5 Fig. Projection of first two slow features for plasmepsin-II.

(A) Time trace SF1 in the training data. (B) Time traced of SF1 in SFA-metadynamics. (C) Time trace of SF2 in the training data. (D) Time trace of SF2 in SFA-metadynamics. Metadynamics simulations using slow features as CVs manage to capture multiple recrossing within a few hundreds of nanoseconds (C, D).
