# Supplementary material for: AlphaFold-SFA: Accelerated sampling of cryptic pocket opening, protein-ligand binding and allostery by AlphaFold, slow feature analysis and metadynamics
Source: PLoS One. 2024 Aug 27;19(8):e0307226. doi: 10.1371/journal.pone.0307226 (PMC11349229; doi:10.1371/journal.pone.0307226)
Supplement: S6 Fig — (A-E) Reweighted free energy surfaces from well-tempered metadynamics projected along Trp41 χ1 and χ2 angles at different time intervals (A-E) highlighted convergence of our metadynamics simulations. (F) Time trace of the reweighting factor rc(t) also highlighted the convergence of metadynamics. It is important to highlight that the reweighting factor reached an asymptotic plateau at the end of the metadynamics simulations which highlights the convergence of the simulation. (PDF) [file pone.0307226.s006.pdf]

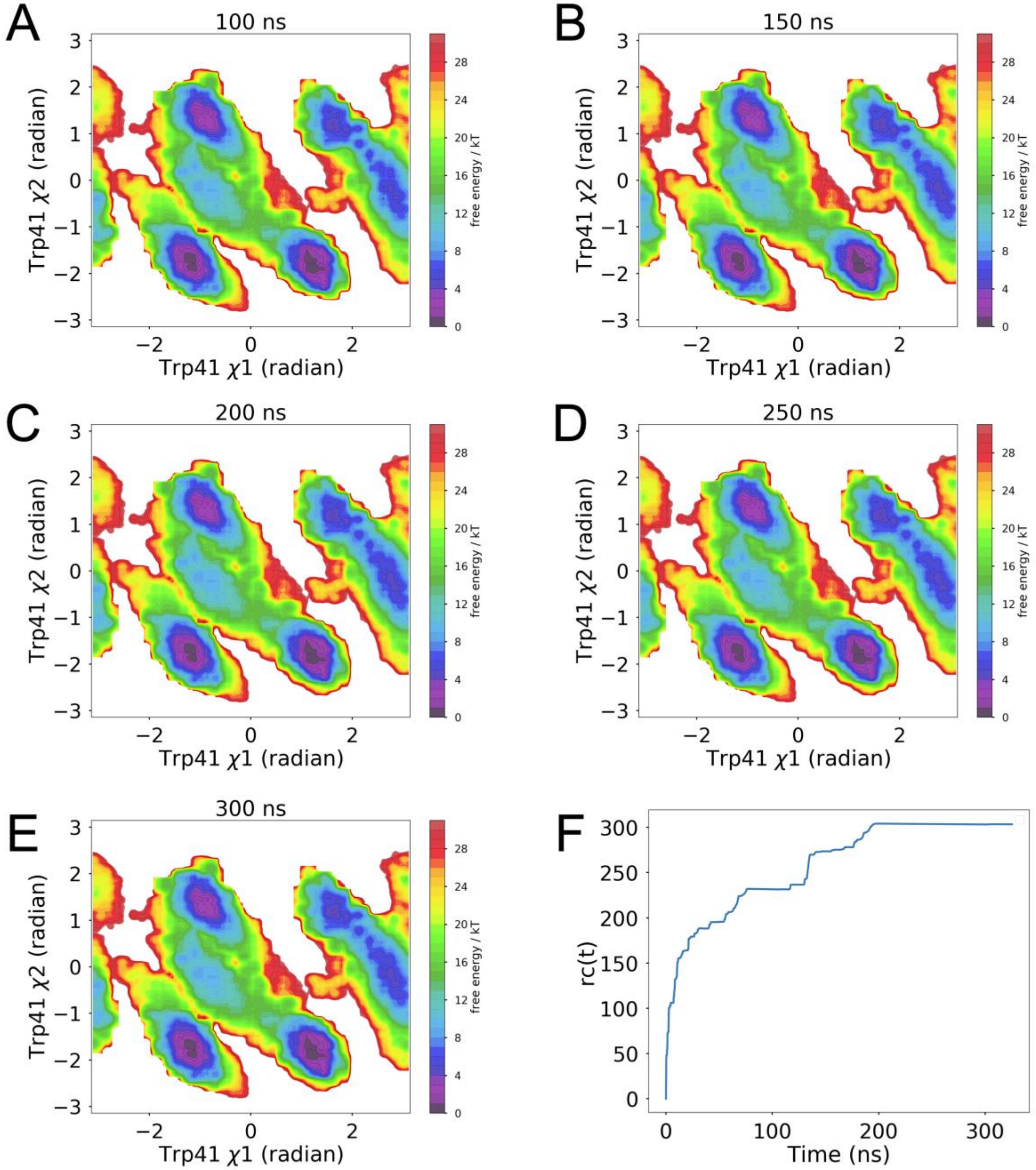

S6 Fig. Converge of SFA-metadynamics simulation in plasmepsin-II.

(A-E) Reweighted free energy surfaces from well-tempered metadynamics projected along Trp41 $\chi1$ and $\chi2$ angles at different time intervals (A-E) highlighted convergence of our metadynamics simulations. (F) Time trace of the reweighting factor *rc(t)* also highlighted the convergence of metadynamics. It is important to highlight that the reweighting factor reached an asymptotic plateau at the end of the metadynamics simulations which highlights the convergence of the simulation.
