# Supplementary material for: AlphaFold-SFA: Accelerated sampling of cryptic pocket opening, protein-ligand binding and allostery by AlphaFold, slow feature analysis and metadynamics
Source: PLoS One. 2024 Aug 27;19(8):e0307226. doi: 10.1371/journal.pone.0307226 (PMC11349229; doi:10.1371/journal.pone.0307226)
Supplement: S7 Fig — (A) Space fill representation of binding pocket in closed plasmepsin-II (PDB: 1LF4). (B) Space fill representation of deep cryptic pocket opening (open, PDB: 2BJU) increases the volume of the binding pocket compared to closed conformation of plasmepsin-II. CASTp server (http://sts.bioe.uic.edu/castp/index.html?3trg) predicts the volume of open and closed conformation are 1185.592 and 391.228 Å3 respectively. (PDF) [file pone.0307226.s007.pdf]

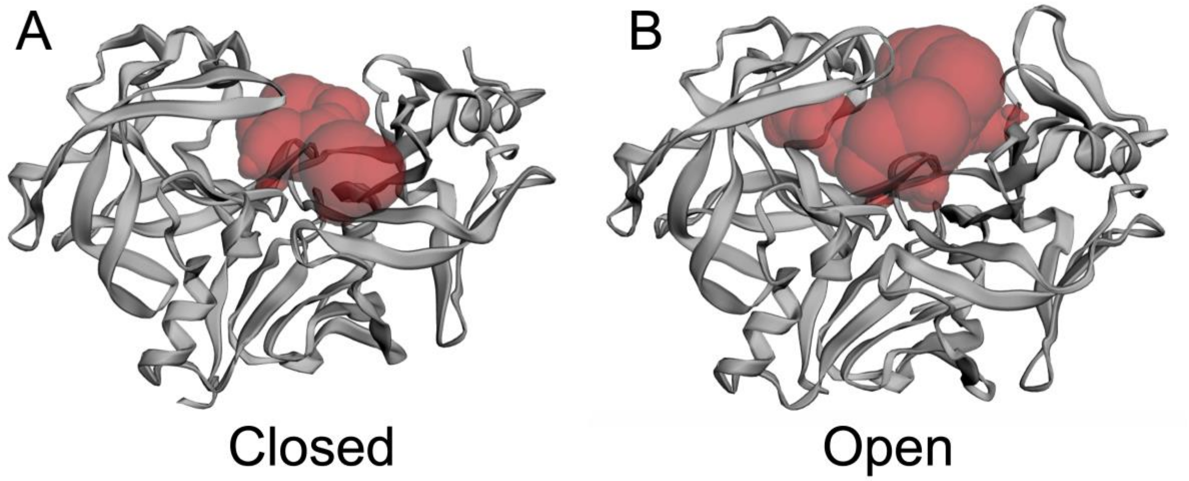

S7 Fig. Volume of cryptic pocket in plasmepsin-II.

(A) Space fill representation of binding pocket in closed plasmepsin-II (PDB: 1LF4). (B) Space fill representation of deep cryptic pocket opening (open, PDB: 2BJU) increases the volume of the binding pocket compared to closed conformation of plasmepsin-II. CASTp server (http://sts.bioe.uic.edu/castp/index.html?3trg) predicts the volume of open and closed conformation are 1185.592 and 391.228 Å^3 respectively.
