# Supplementary material for: AlphaFold-SFA: Accelerated sampling of cryptic pocket opening, protein-ligand binding and allostery by AlphaFold, slow feature analysis and metadynamics
Source: PLoS One. 2024 Aug 27;19(8):e0307226. doi: 10.1371/journal.pone.0307226 (PMC11349229; doi:10.1371/journal.pone.0307226)
Supplement: S8 Fig — (A) Free energy surface projected along flap opening and Tyr77 χ1 angle for unbiased MD simulations starting from closed state. (B) Reweighted free energy surface projected along flap opening and Tyr77 χ1 angle from SFA-metadynamics starting from closed state. (C) Free energy surface projected along flap opening and Tyr77 χ1 angle for unbiased MD simulations starting from open state. (D) Reweighted free energy surface projected along flap opening and Tyr77 χ1 angle from SFA-metadynamics starting from open state. SFA-metadynamics captures flipping of Tyr77 χ1 angle and flap opening within few hundreds of nanoseconds when compared to 16 μs of unbiased molecular dynamics simulation of started from closed and open states respectively. AlphaFold generated conformations are shown as black dots. (PDF) [file pone.0307226.s008.pdf]

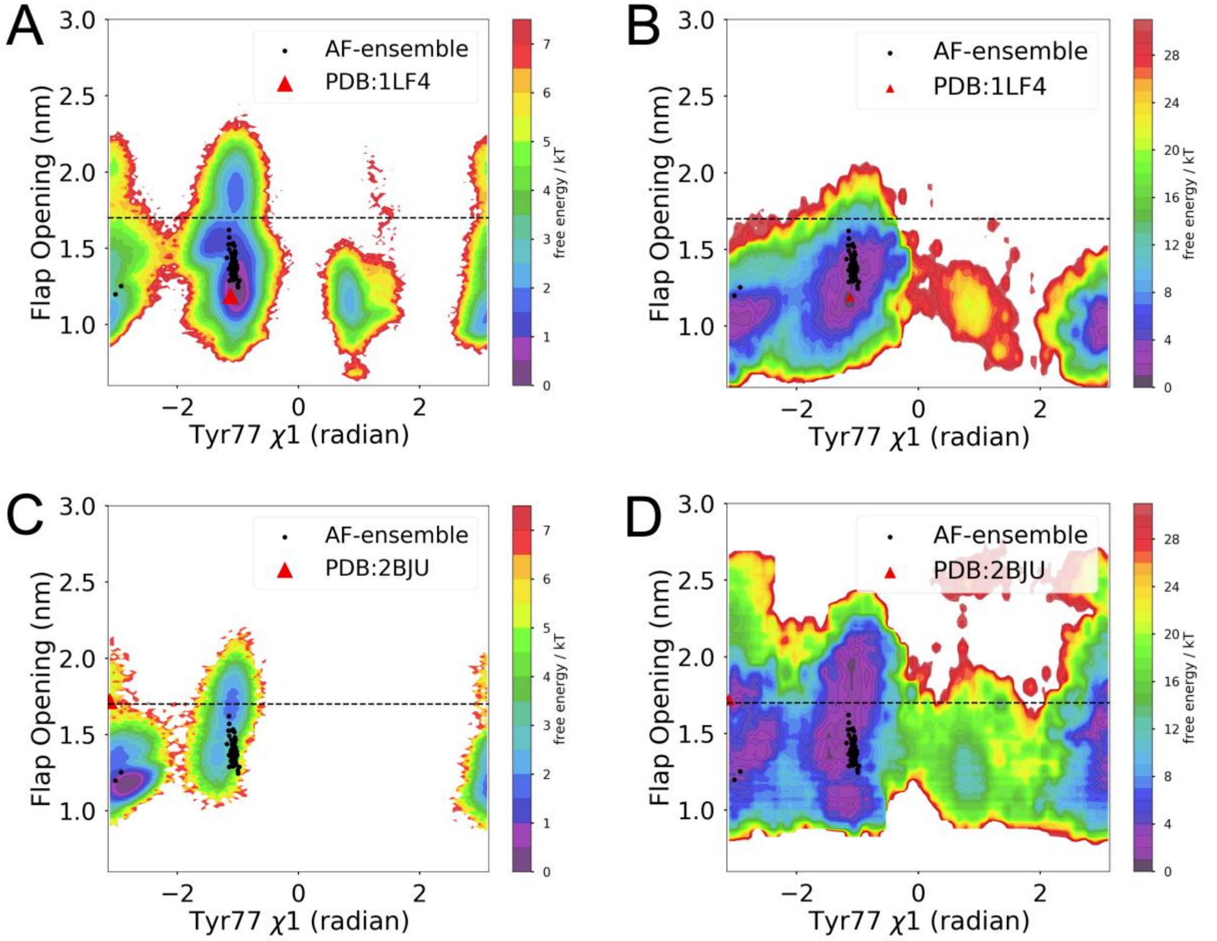

S8 Fig. Reweighted free energy surface captures flap opening and flipping of Tyr77 in plasmepsin-II.

(A) Free energy surface projected along flap opening and Tyr77 $\chi 1$ angle for unbiased MD simulations starting from closed state. (B) Reweighted free energy surface projected along flap opening and Tyr77 $\chi 1$ angle from SFA-metadynamics starting from closed state. (C) Free energy surface projected along flap opening and Tyr77 $\chi 1$ angle for unbiased MD simulations starting from open state. (D) Reweighted free energy surface projected along flap opening and Tyr77 $\chi 1$ angle from SFA-metadynamics starting from open state. SFA-metadynamics captures flipping of Tyr77 $\chi 1$ angle and flap opening within few hundreds of nanoseconds when compared to 16 μs of unbiased molecular dynamics simulation of started from closed and open states respectively. AlphaFold generated conformations are shown as black dots.
