# Supplementary material for: AlphaFold-SFA: Accelerated sampling of cryptic pocket opening, protein-ligand binding and allostery by AlphaFold, slow feature analysis and metadynamics
Source: PLoS One. 2024 Aug 27;19(8):e0307226. doi: 10.1371/journal.pone.0307226 (PMC11349229; doi:10.1371/journal.pone.0307226)
Supplement: S9 Fig — (A) Conformation of cryptic pocket open state with Trp41 χ2 angle of +1 radian (orange, PDB: 2BJU). (B) Conformation of cryptic pocket open state with Trp41 χ2 angle of -1 radian (blue, PDB: 4Z22). (C) Reweighted free energy surface from SFA-metadynamics along Trp41 χ1 and χ2 angles highlighted alternate states (A, B) associated with χ2 flipping. AlphaFold ensembles are highlighted in black dots. It is important to note that AlphaFold failed to sample an alternate Trp41 χ2 angle of -1 radian. The black dotted arrows highlight the flipping of Trp41 and the flap opening. (PDF) [file pone.0307226.s009.pdf]

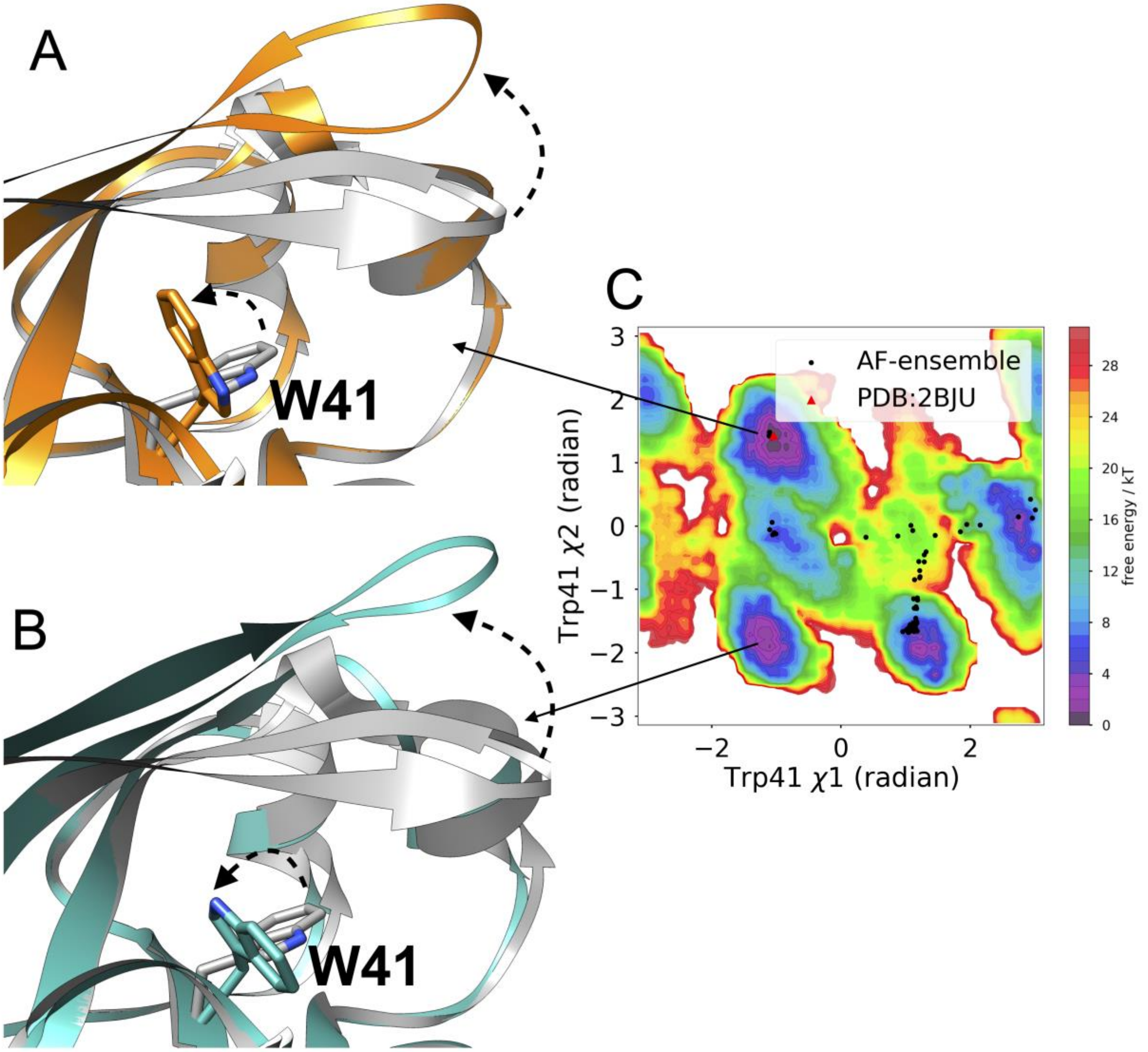

S9 Fig. SFA-metadynamics samples alternative cryptic pocket open states in plasmepsin-II.
(A) Conformation of cryptic pocket *open* state with Trp41 $\chi 2$ angle of +1 radian (orange, PDB: 2BJU). (B) Conformation of cryptic pocket *open* state with Trp41 $\chi 2$ angle of -1 radian (blue, PDB: 4Z22). (C) Reweighted free energy surface from SFA-metadynamics along Trp41 $\chi 1$ and $\chi 2$ angles highlighted alternate states (A, B) associated with $\chi 2$ flipping. AlphaFold ensembles are highlighted in black dots. It is important to note that AlphaFold failed to sample an alternate Trp41 $\chi 2$ angle of -1 radian. The black dotted arrows highlight the flipping of Trp41 and the flap opening.
