# Supplementary material for: AlphaFold-SFA: Accelerated sampling of cryptic pocket opening, protein-ligand binding and allostery by AlphaFold, slow feature analysis and metadynamics
Source: PLoS One. 2024 Aug 27;19(8):e0307226. doi: 10.1371/journal.pone.0307226 (PMC11349229; doi:10.1371/journal.pone.0307226)
Supplement: S10 Fig — (A) Snapshot from traditional funnel metadynamics (blue) highlights the formation of H-bond interaction between Tyr77 and Asp34 which blocks deep cryptic pocket in plasmepsin-II when compared to the starting conformation (magenta, PDB: 7QYH). (B) Time trace of H-bond interaction between Tyr77-Asp34 in traditional funnel metadynamics. (C) Time trace of H-bond interaction between Tyr77-Asp34 in SFA-augmented funnel metadynamics. (PDF) [file pone.0307226.s010.pdf]

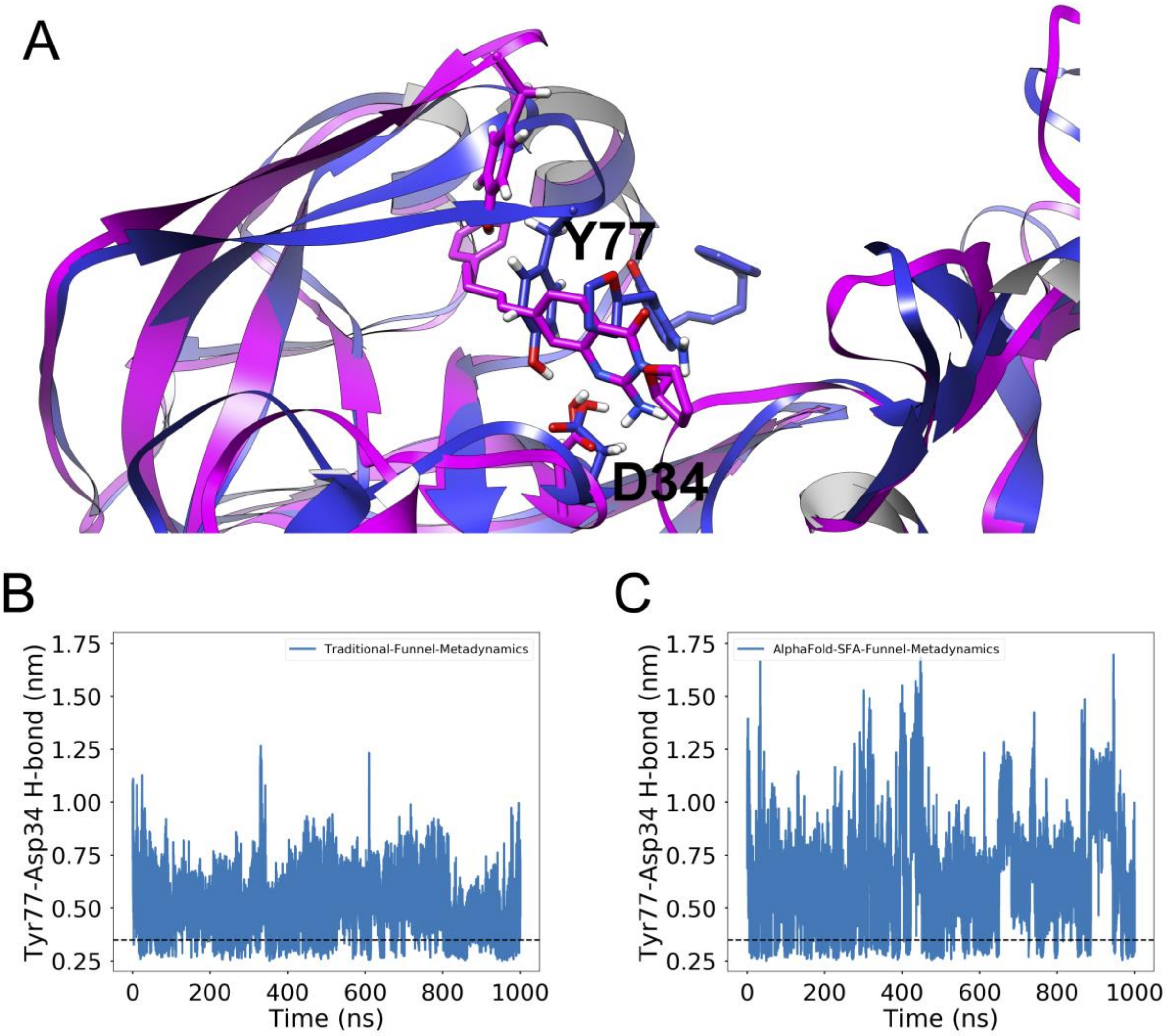

S10 Fig. SFA-augmented funnel metadynamics captures protein dynamics of plasmepsin-II.
(A) Snapshot from traditional funnel metadynamics (blue) highlights the formation of H-bond interaction between Tyr77 and Asp34 which blocks deep cryptic pocket in plasmepsin-II when compared to the starting conformation (magenta, PDB: 7QYH). (B) Time trace of H-bond interaction between Tyr77-Asp34 in traditional funnel metadynamics. (C) Time trace of H-bond interaction between Tyr77-Asp34 in SFA-augmented funnel metadynamics.
