# Supplementary material for: AlphaFold-SFA: Accelerated sampling of cryptic pocket opening, protein-ligand binding and allostery by AlphaFold, slow feature analysis and metadynamics
Source: PLoS One. 2024 Aug 27;19(8):e0307226. doi: 10.1371/journal.pone.0307226 (PMC11349229; doi:10.1371/journal.pone.0307226)
Supplement: S11 Fig — (A) SF1 weights and corresponding sin/cos transformed dihedral angles. (B) SF2 weights and corresponding sin/cos transformed dihedral angles (https://github.com/sbhakat/AlphaFold-SFA/blob/main/RIPK2/feature-lists.ipynb) associated with amino acid residues in RIPK2. (PDF) [file pone.0307226.s011.pdf]

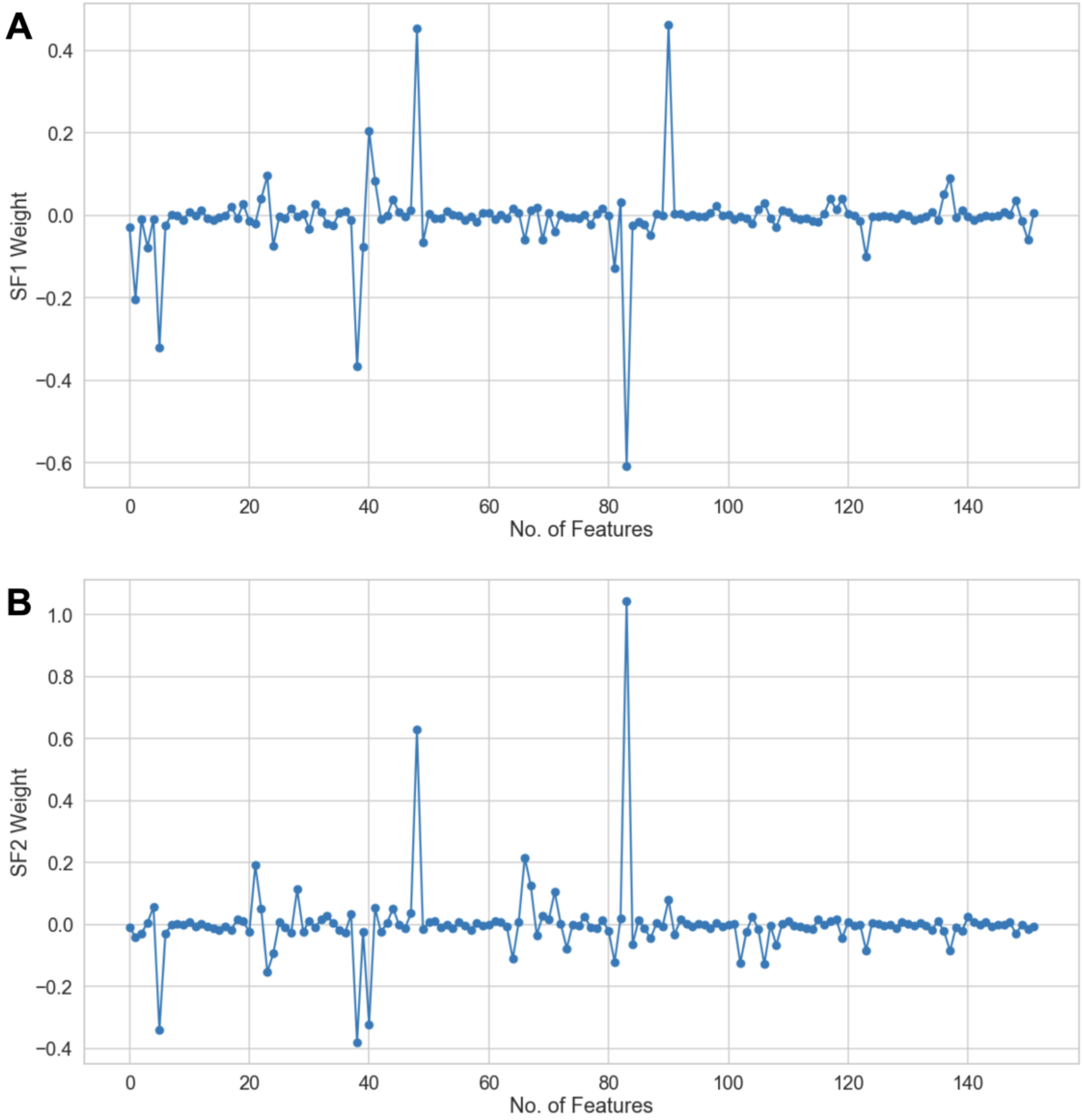

S11 Fig. SFA weights and corresponding features in RIPK2.

(A) SF1 weights and corresponding sin/cos transformed dihedral angles. (B) SF2 weights and corresponding sin/cos transformed dihedral angles (https://github.com/sbhakat/AlphaFold-SFA/blob/main/RIPK2/feature-lists.ipynb) associated with amino acid residues in RIPK2.
