# Supplementary material for: AlphaFold-SFA: Accelerated sampling of cryptic pocket opening, protein-ligand binding and allostery by AlphaFold, slow feature analysis and metadynamics
Source: PLoS One. 2024 Aug 27;19(8):e0307226. doi: 10.1371/journal.pone.0307226 (PMC11349229; doi:10.1371/journal.pone.0307226)
Supplement: S12 Fig — (A) Time trace SF1 in the training data. (B) Time traced of SF1 in SFA-metadynamics. (C) Time trace of SF2 in the training data. (D) Time trace of SF2 in SFA-metadynamics. Metadynamics accelerated the sampling along first two slow features which enabled sampling of allosteric dynamics in RIPK2. (PDF) [file pone.0307226.s012.pdf]

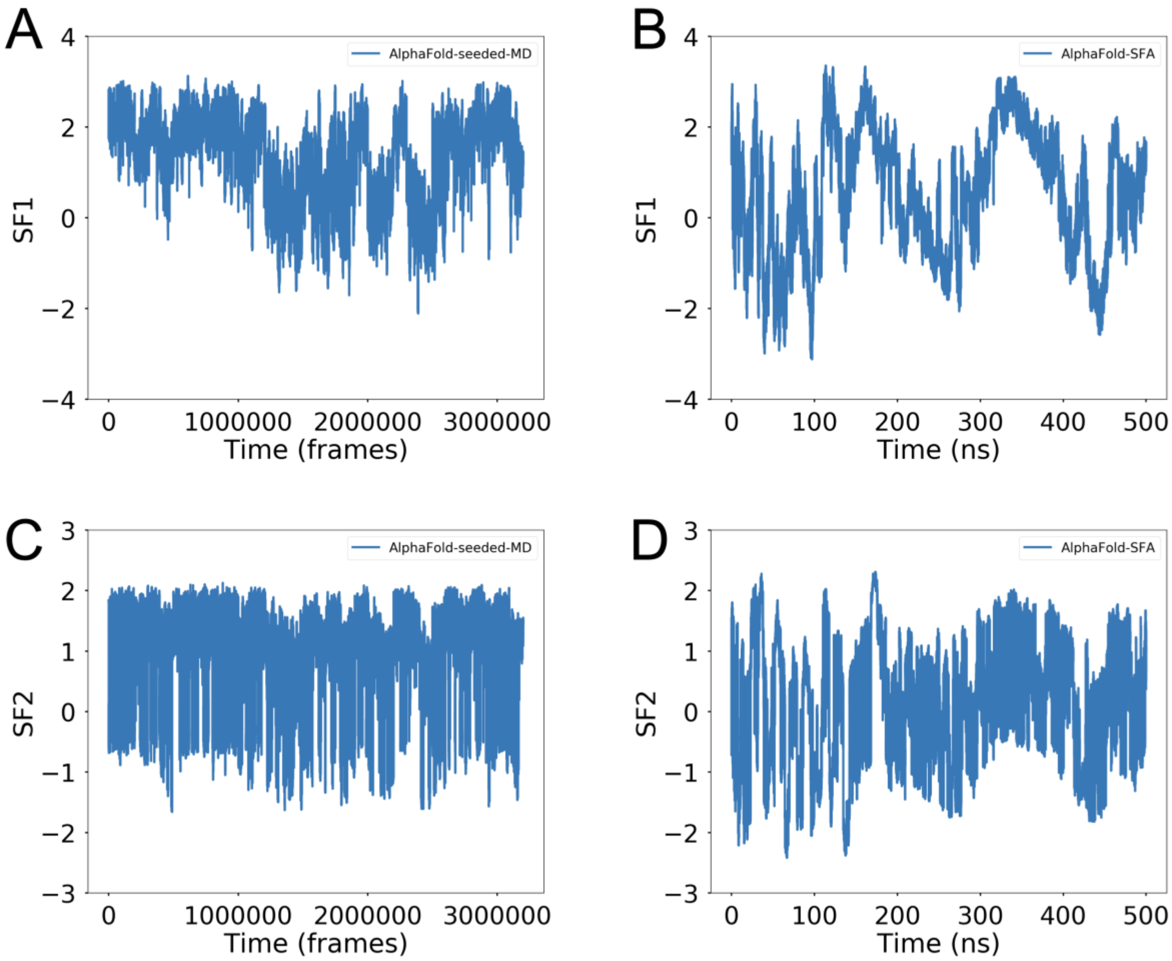

S12 Fig. Sampling of first two slow features in the training data and metadynamics simulations starting with apo RIPK2.

(A) Time trace SF1 in the training data. (B) Time traced of SF1 in SFA-metadynamics. (C) Time trace of SF2 in the training data. (D) Time trace of SF2 in SFA-metadynamics. Metadynamics accelerated the sampling along first two slow features which enabled sampling of allosteric dynamics in RIPK2.
