# Supplementary material for: AlphaFold-SFA: Accelerated sampling of cryptic pocket opening, protein-ligand binding and allostery by AlphaFold, slow feature analysis and metadynamics
Source: PLoS One. 2024 Aug 27;19(8):e0307226. doi: 10.1371/journal.pone.0307226 (PMC11349229; doi:10.1371/journal.pone.0307226)
Supplement: S13 Fig — (A-D) Reweighted free energy surfaces from well-tempered SFA-metadynamics projected along Phe165 χ1 and χ2 angles at different time intervals highlighted convergence of the simulation. (PDF) [file pone.0307226.s013.pdf]

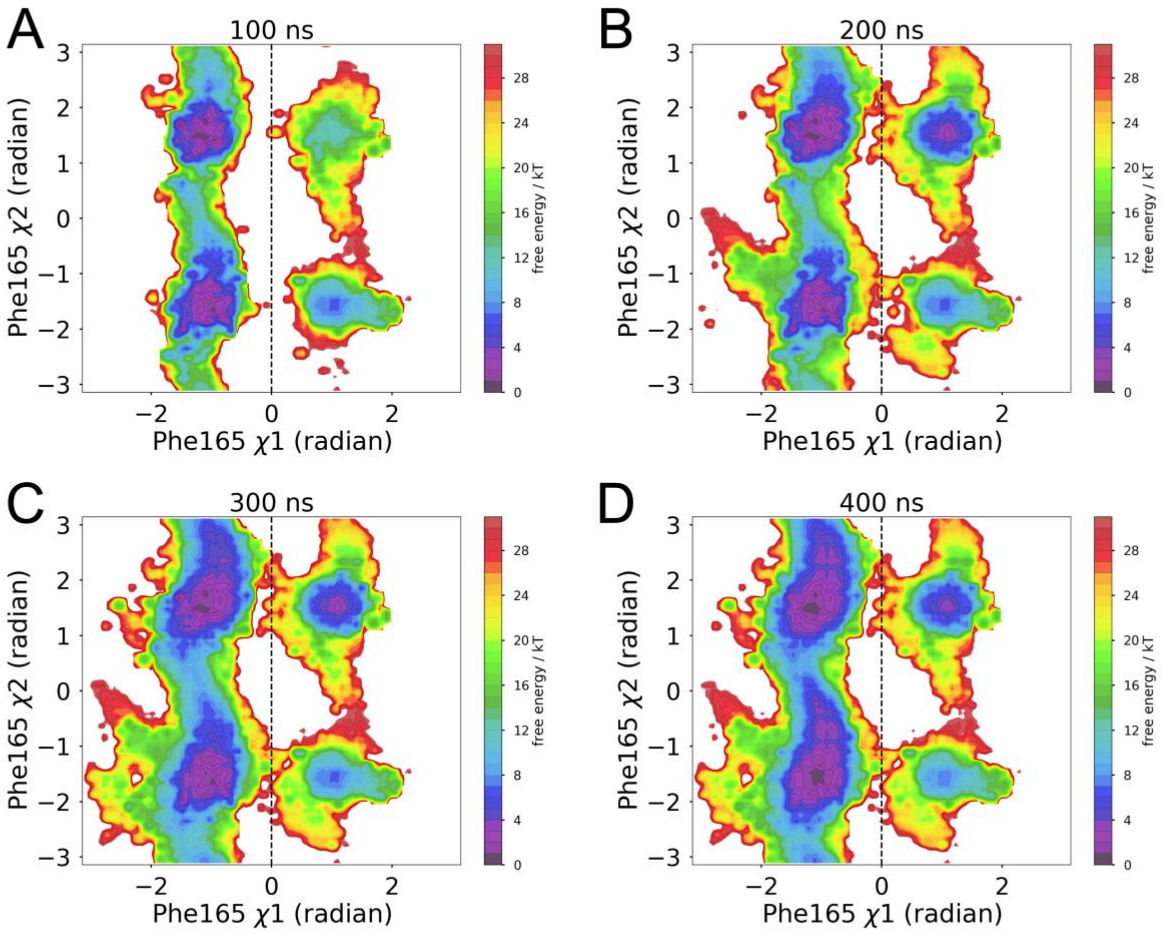

S13 Fig. Convergence of SFA metadynamics starting with apo RIPK2.

(A-D) Reweighted free energy surfaces from well-tempered SFA-metadynamics projected along Phe165 $\chi 1$ and $\chi 2$ angles at different time intervals highlighted convergence of the simulation.
