# Supplementary material for: AlphaFold-SFA: Accelerated sampling of cryptic pocket opening, protein-ligand binding and allostery by AlphaFold, slow feature analysis and metadynamics
Source: PLoS One. 2024 Aug 27;19(8):e0307226. doi: 10.1371/journal.pone.0307226 (PMC11349229; doi:10.1371/journal.pone.0307226)
Supplement: S14 Fig — Distribution of Phe165 dihedral angles in RIPK2 crystal structures available in RCSB PDB projected on reweighted free energy surface from SFA-metadynamics. Inactive RIPK2 corresponds to PDB: 5J7B, 5AR4, 6SZJ and 6FU5. (PDF) [file pone.0307226.s014.pdf]

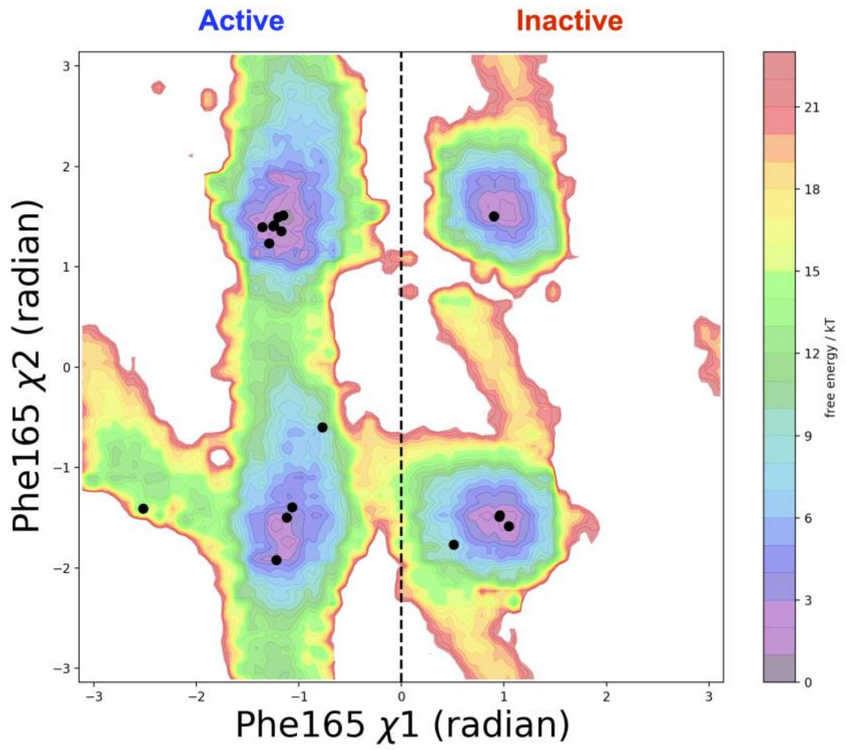

S14 Fig. SFA metadynamics samples active and inactive conformation of RIPK2.
Distribution of Phe165 dihedral angles in RIPK2 crystal structures available in RCSB PDB projected on reweighted free energy surface from SFA-metadynamics. Inactive RIPK2 corresponds to PDB: 5J7B, 5AR4, 6SZJ and 6FU5.
