# Supplementary material for: AlphaFold-SFA: Accelerated sampling of cryptic pocket opening, protein-ligand binding and allostery by AlphaFold, slow feature analysis and metadynamics
Source: PLoS One. 2024 Aug 27;19(8):e0307226. doi: 10.1371/journal.pone.0307226 (PMC11349229; doi:10.1371/journal.pone.0307226)
Supplement: S15 Fig — (A) Time trace of Arg65—Ser168 distance during SFA-metadynamics. (B) Time trace of Arg65—Ser168 distance in unbiased MD simulation of apo RIPK2. SFA-metadynamics managed to sample multiple transitions between active and inactive states of RIPK2 (demarked by the dashed line at 0.82 nm) compared to unbiased MD simulation which remained in the active state. (PDF) [file pone.0307226.s015.pdf]

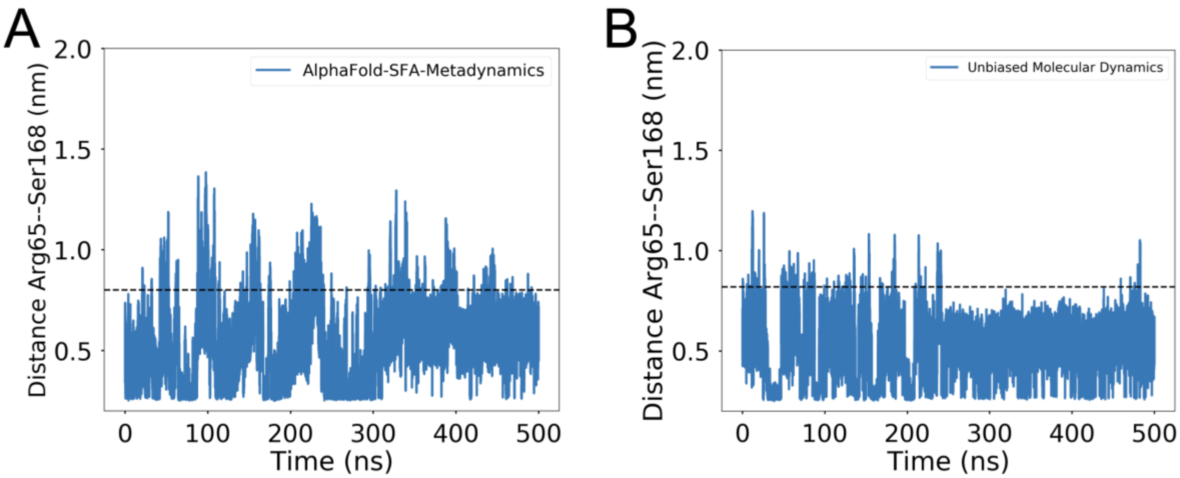

S15 Fig. SFA-metadynamics samples multiple transitions associated with H-bond involving Arg65 and Ser168.

(A) Time trace of Arg65—Ser168 distance during SFA-metadynamics. (B) Time trace of Arg65—Ser168 distance in unbiased MD simulation of apo RIPK2. SFA-metadynamics managed to sample multiple transitions between active and inactive states of RIPK2 (demarked by the dashed line at 0.82 nm) compared to unbiased MD simulation which remained in the active state.
