# Supplementary material for: AlphaFold-SFA: Accelerated sampling of cryptic pocket opening, protein-ligand binding and allostery by AlphaFold, slow feature analysis and metadynamics
Source: PLoS One. 2024 Aug 27;19(8):e0307226. doi: 10.1371/journal.pone.0307226 (PMC11349229; doi:10.1371/journal.pone.0307226)
Supplement: S16 Fig — (A) Time trace of RMSD of αC helix in unbiased MD. (B) Time trace of RMSD of αC helix in SFA-metadynamics simulation. In unbiased MD simulations αC helix remained in an inward conformation due to H-bond interaction involving Arg65—Ser168. SFA-metadynamics simulations managed to capture conformational dynamics associated with the activation loop of RIPK2 which sampled flipping of Trp170. Flipping of Trp170 destabilizes the activation loop which breaks Arg65—Ser168 interaction and resulted in an outward conformation of αC helix. (PDF) [file pone.0307226.s016.pdf]

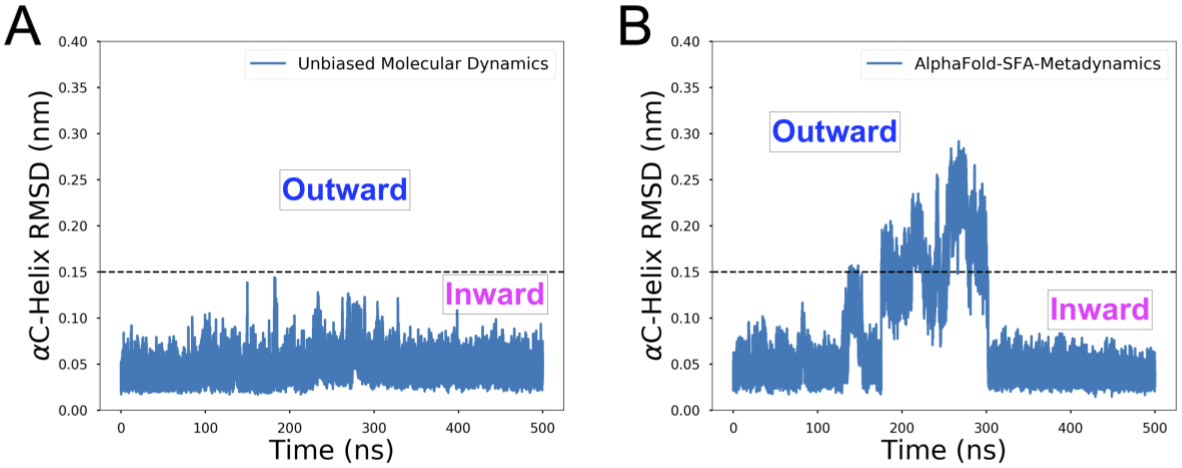

S16 Fig. SFA metadynamics captures inward to outward transition in apo RIPK2.

(A) Time trace of RMSD of αC helix in unbiased MD. (B) Time trace of RMSD of αC helix in SFA-metadynamics simulation. In unbiased MD simulations αC helix remained in an *inward* conformation due to H-bond interaction involving Arg65—Ser168. SFA-metadynamics simulations managed to capture conformational dynamics associated with the activation loop of RIPK2 which sampled flipping of Trp170. Flipping of Trp170 destabilizes the activation loop which breaks Arg65—Ser168 interaction and resulted in an *outward* conformation of αC helix.
