# Supplementary material for: AlphaFold-SFA: Accelerated sampling of cryptic pocket opening, protein-ligand binding and allostery by AlphaFold, slow feature analysis and metadynamics
Source: PLoS One. 2024 Aug 27;19(8):e0307226. doi: 10.1371/journal.pone.0307226 (PMC11349229; doi:10.1371/journal.pone.0307226)
Supplement: S17 Fig — (A) Free energy surface projected along χ1 and χ2 angles of Trp170 from unbiased MD simulation of RIPK2+XIAP complex. (B) Free energy surface projected along χ1 and χ2 angles of Phe165 from unbiased MD simulation of RIPK2+XIAP complex. (C) Time trace of Arg65—Ser168 distance in unbiased MD simulation of RIPK2+XIAP complex. (D) Time trace of Glu68—Trp170 distance in unbiased MD simulation of RIPK2+XIAP complex. (E) Time trace of Arg171—XIAP-Asp214 distance in unbiased MD simulation of RIPK2+XIAP complex. (PDF) [file pone.0307226.s017.pdf]

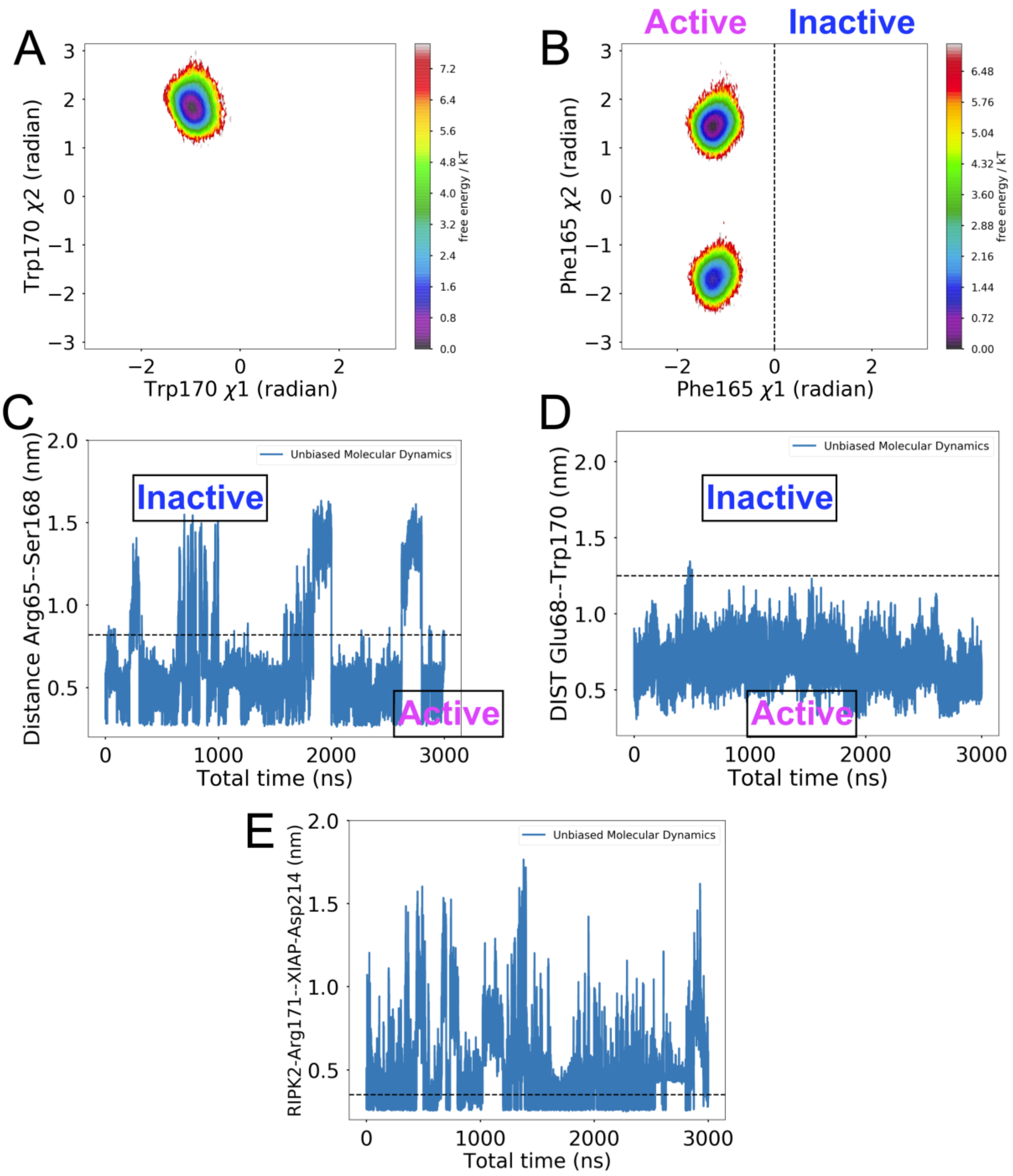

S17 Fig. RIPK2-XIAP complex stabilizes the active state of RIPK2.

(A) Free energy surface projected along $\chi1$ and $\chi2$ angles of Trp170 from unbiased MD simulation of RIPK2+XIAP complex. (B) Free energy surface projected along $\chi1$ and $\chi2$ angles of Phe165 from unbiased MD simulation of RIPK2+XIAP complex. (C) Time trace of Arg65—Ser168 distance in unbiased MD simulation of RIPK2+XIAP complex. (D) Time trace of Glu68—Trp170 distance in unbiased MD simulation of RIPK2+XIAP complex. (E) Time trace of Arg171—XIAP-Asp214 distance in unbiased MD simulation of RIPK2+XIAP complex.
