# Supplementary material for: AlphaFold-SFA: Accelerated sampling of cryptic pocket opening, protein-ligand binding and allostery by AlphaFold, slow feature analysis and metadynamics
Source: PLoS One. 2024 Aug 27;19(8):e0307226. doi: 10.1371/journal.pone.0307226 (PMC11349229; doi:10.1371/journal.pone.0307226)
Supplement: S18 Fig — It highlights orientation of Phe165, Trp170, Arg65 and Ser168, key residues involved in conformational dynamics of RIPK2. (PDF) [file pone.0307226.s018.pdf]

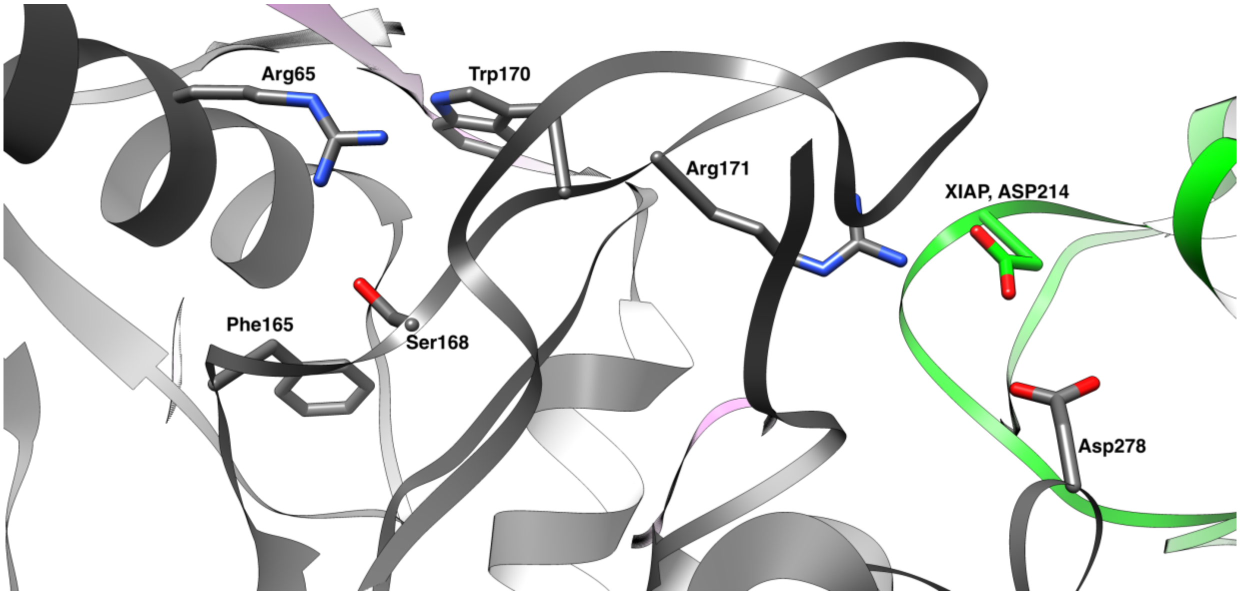

S18 Fig. **Structural insights of how Arg171 present in the activation loop of RIPK2 interacts with the Asp214 of XIAP (green)**.

It highlights orientation of Phe165, Trp170, Arg65 and Ser168, key residues involved in conformational dynamics of RIPK2.
