# Supplementary material for: AlphaFold-SFA: Accelerated sampling of cryptic pocket opening, protein-ligand binding and allostery by AlphaFold, slow feature analysis and metadynamics
Source: PLoS One. 2024 Aug 27;19(8):e0307226. doi: 10.1371/journal.pone.0307226 (PMC11349229; doi:10.1371/journal.pone.0307226)
Supplement: S19 Fig — Sampling of Phe165 (A), Trp170 (B) and Arg65 (C) in AlphaFold generated conformational ensemble of apo RIPK2. (PDF) [file pone.0307226.s019.pdf]

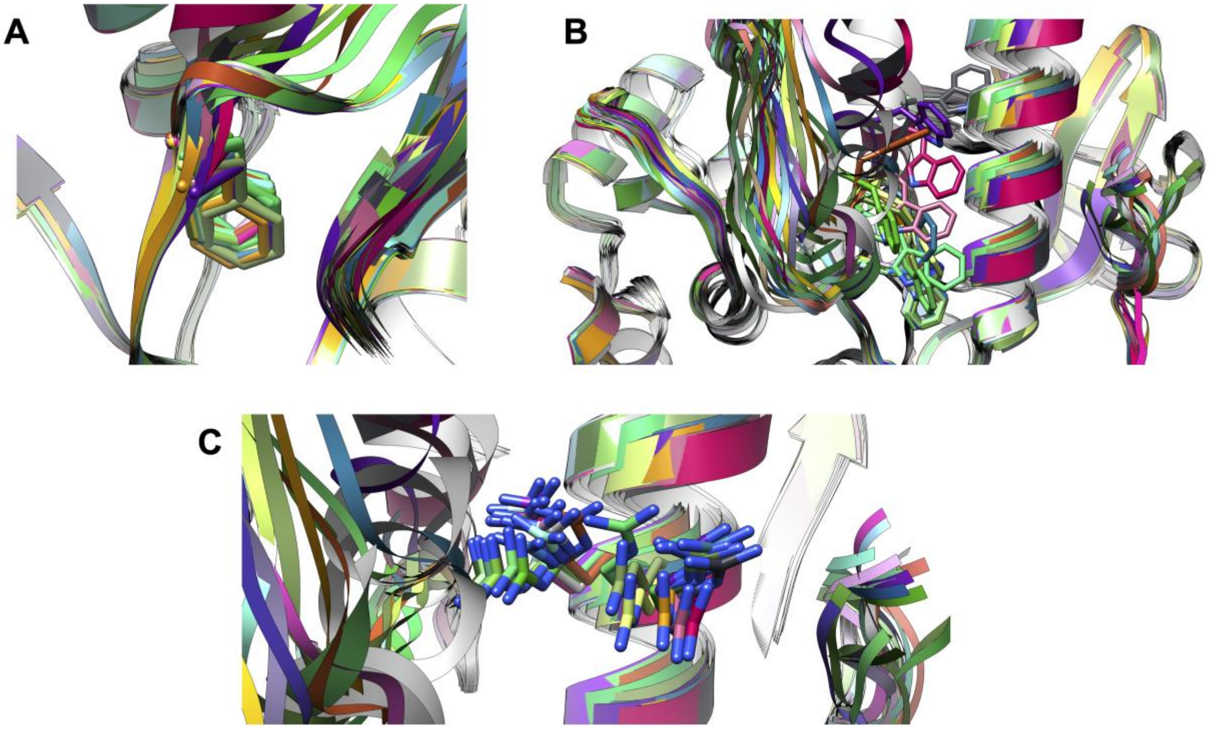

S19 Fig. Conformational sampling in AlphaFold generated ensemble of apo RIPK2.
Sampling of Phe165 (A), Trp170 (B) and Arg65 (C) in AlphaFold generated conformational ensemble of apo RIPK2.
