# Supplementary material for: AlphaFold-SFA: Accelerated sampling of cryptic pocket opening, protein-ligand binding and allostery by AlphaFold, slow feature analysis and metadynamics
Source: PLoS One. 2024 Aug 27;19(8):e0307226. doi: 10.1371/journal.pone.0307226 (PMC11349229; doi:10.1371/journal.pone.0307226)
Supplement: S20 Fig — (A) Crystal structures of holo-BRAF highlights the coupled motion involving DFG-Phe flipping and the ‘outward’ conformation of αC helix. Blue indicates PDB: 4EHG and the magenta indicates PDB: 2FB8. (B) Outward conformation of αC helix breaks the salt bridge interaction between Lys483 and Glu501. Breaking of the salt bridge interaction and flipping of Phe595 is a hall mark of Src-like inactive conformation. (PDF) [file pone.0307226.s020.pdf]

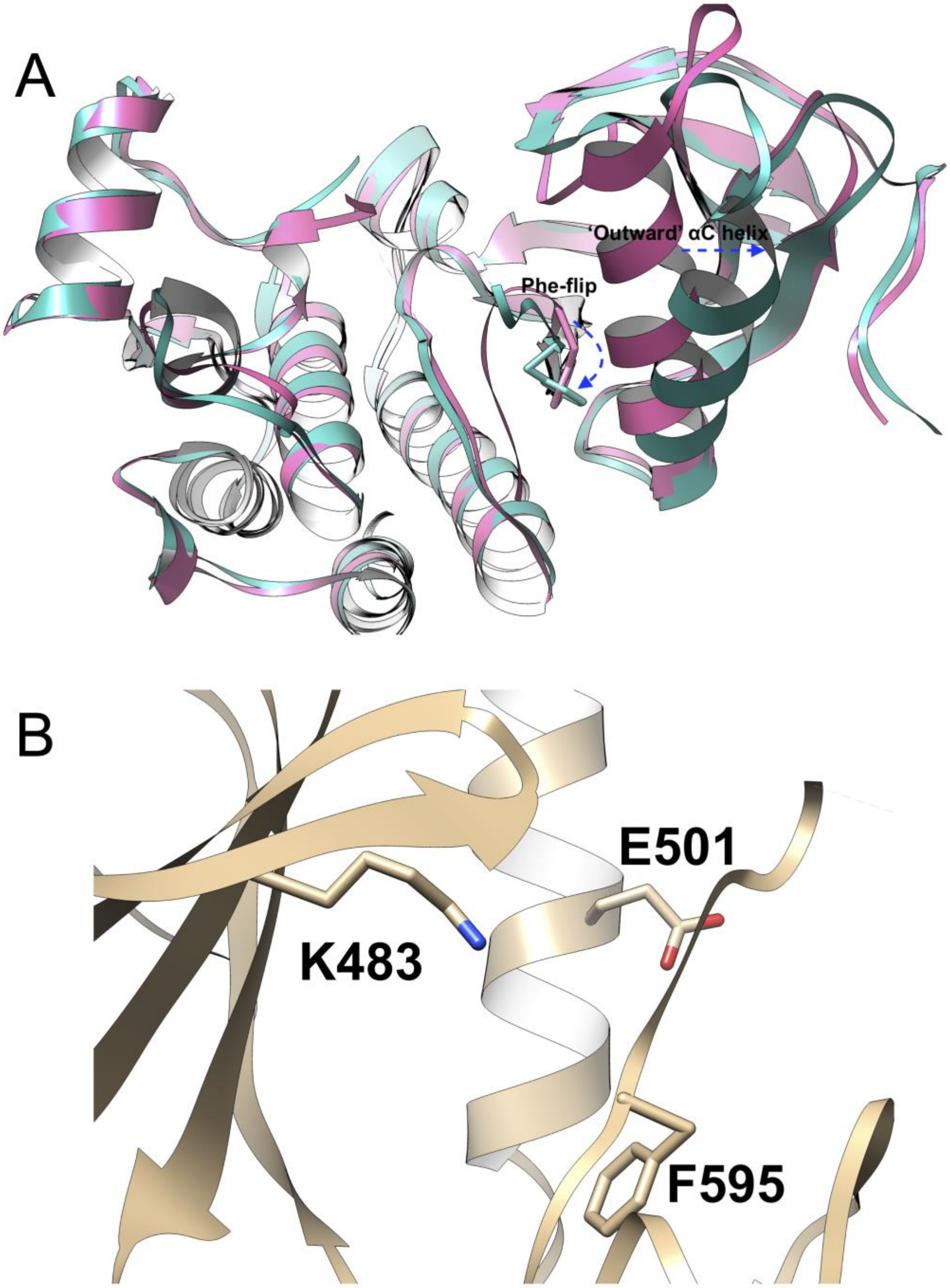

S20 Fig. Active to inactive transition has been sampled in homologous serine-threonine kinase, BRAF.

(A) Crystal structures of holo-BRAF highlights the coupled motion involving DFG-Phe flipping and the ‘outward’ conformation of αC helix. Blue indicates PDB: 4EHG and the magenta indicates PDB: 2FB8. (B) Outward conformation of αC helix breaks the salt bridge interaction between Lys483 and Glu501. Breaking of the salt bridge interaction and flipping of Phe595 is a hall mark of Src-like inactive conformation.
