# Supplementary material for: AlphaFold-SFA: Accelerated sampling of cryptic pocket opening, protein-ligand binding and allostery by AlphaFold, slow feature analysis and metadynamics
Source: PLoS One. 2024 Aug 27;19(8):e0307226. doi: 10.1371/journal.pone.0307226 (PMC11349229; doi:10.1371/journal.pone.0307226)
Supplement: S21 Fig — (A) Time-trace of distance between Lys209 of RIPK2 and Glu211 of XIAP during total 3 μs of unbiased MD simulation of XIAP-RIPK2 complex. The dashed line at 0.35 nm indicates formation of H-bond interaction involving Lys209—Glu211. (B) Orientation of Lys209 and Ile208 of RIPK2 and Glu211 of XIAP is highlighted for visual inspection. (PDF) [file pone.0307226.s021.pdf]

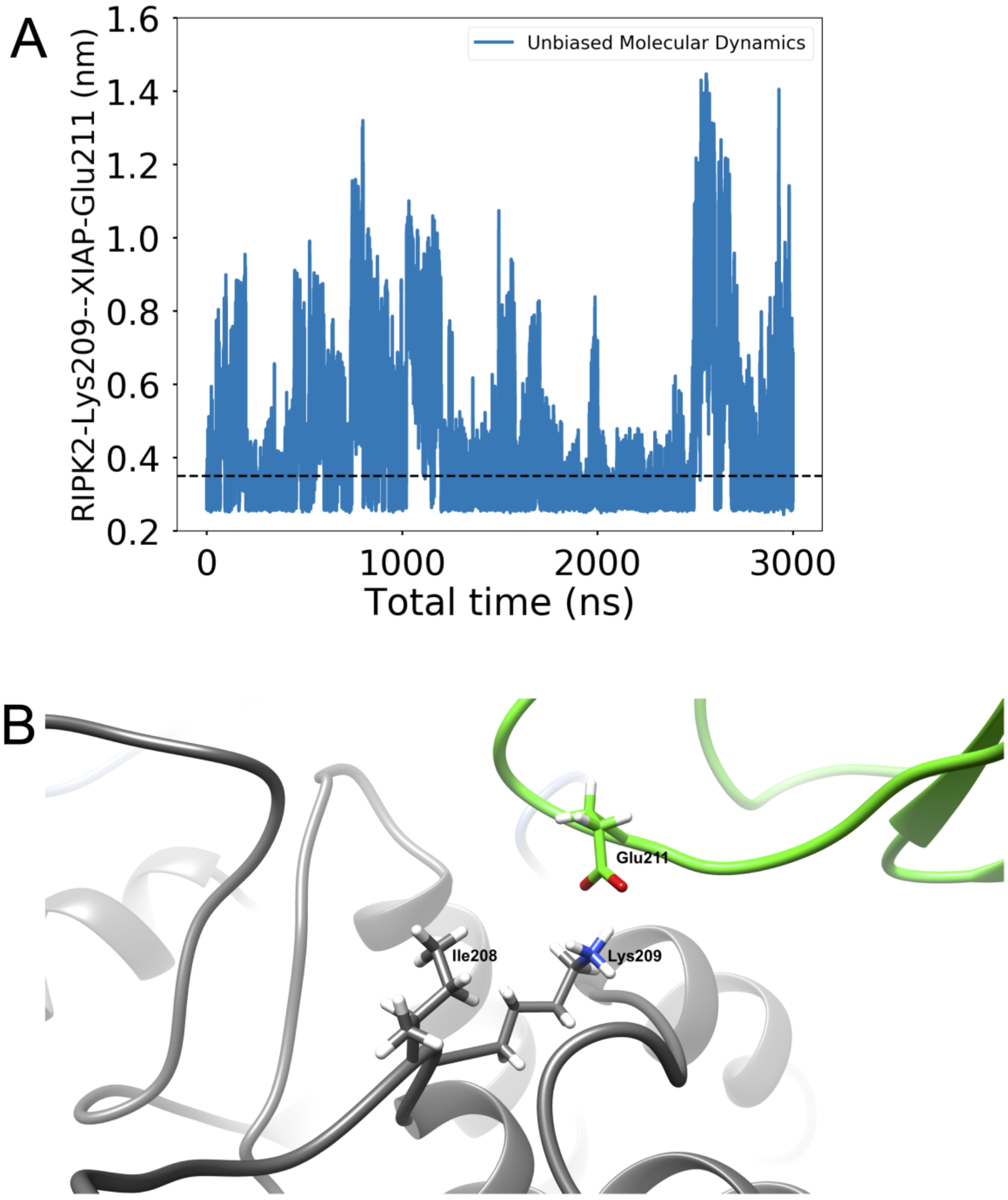

S21 Fig. Interaction between Lys209 of RIPK2 and Glu211 of XIAP is critical for the formation of protein-protein complex.

(A) Time-trace of distance between Lys209 of RIPK2 and Glu211 of XIAP during total 3 μs of unbiased MD simulation of XIAP-RIPK2 complex. The dashed line at 0.35 nm indicates formation of H-bond interaction involving Lys209—Glu211. (B) Orientation of Lys209 and Ile208 of RIPK2 and Glu211 of XIAP is highlighted for visual inspection.
