# Supplementary material for: AlphaFold-SFA: Accelerated sampling of cryptic pocket opening, protein-ligand binding and allostery by AlphaFold, slow feature analysis and metadynamics
Source: PLoS One. 2024 Aug 27;19(8):e0307226. doi: 10.1371/journal.pone.0307226 (PMC11349229; doi:10.1371/journal.pone.0307226)
Supplement: S22 Fig — The activation loop of the RIPK2 is highlighted in red and the XIAP is highlighted in ‘seagreen blue’. (PDF) [file pone.0307226.s022.pdf]

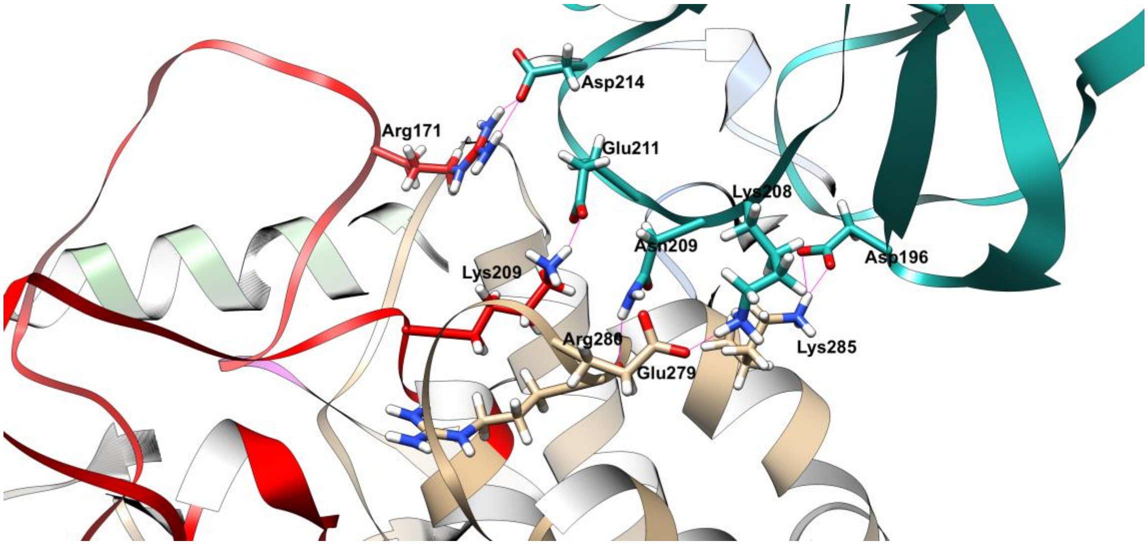

S22 Fig. Protein-protein interface of RIPK2-XIAP complex highlighting key residues involved in H-bond interactions.

The activation loop of the RIPK2 is highlighted in red and the XIAP is highlighted in ‘seagreen blue’.
