# Supplementary material for: AlphaFold-SFA: Accelerated sampling of cryptic pocket opening, protein-ligand binding and allostery by AlphaFold, slow feature analysis and metadynamics
Source: PLoS One. 2024 Aug 27;19(8):e0307226. doi: 10.1371/journal.pone.0307226 (PMC11349229; doi:10.1371/journal.pone.0307226)
Supplement: S23 Fig — (A) Time trace of RIPK2-Arg280—XIAP-Asn209 distance. (B) Time trace of RIPK2-Lys285—XIAP-Asp196 distance. (C) Time trace of RIPK2-Glu279—XIAP-Lys208 distance. Temporal evolution of hydrogen bond distances throughout molecular dynamics simulations, encompassing 15 independent runs each lasting 200 nanoseconds, across crucial residues that play a role in stabilizing the RIPK2-XIAP complex. The dotted line at 0.35 nm indicates complete formation of H-bond. (PDF) [file pone.0307226.s023.pdf]

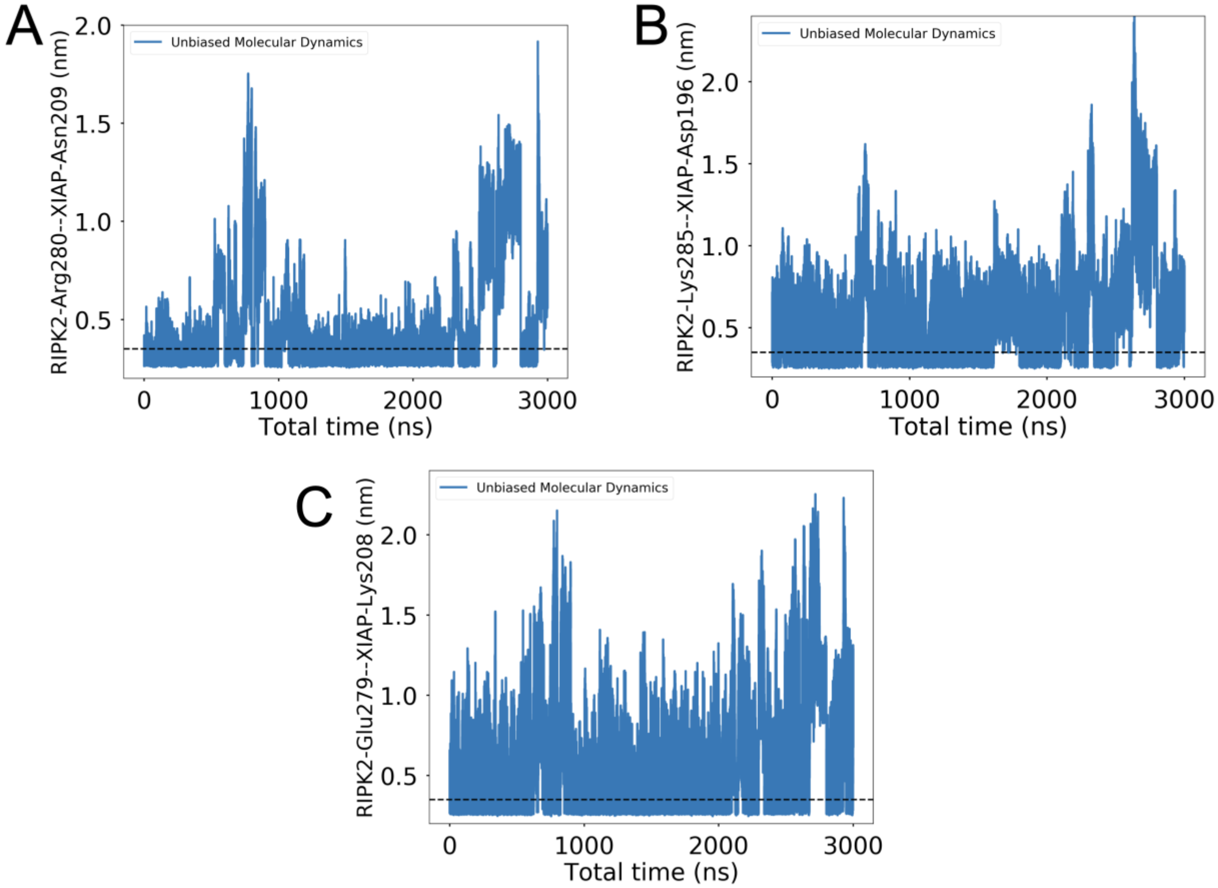

S23 Fig. Time trace of key interactions involving the interface of RIPK2-XIAP complex during unbiased MD simulations.

(A) Time trace of RIPK2-Arg280—XIAP-Asn209 distance. (B) Time trace of RIPK2-Lys285—XIAP-Asp196 distance. (C) Time trace of RIPK2-Glu279—XIAP-Lys208 distance. Temporal evolution of hydrogen bond distances throughout molecular dynamics simulations, encompassing 15 independent runs each lasting 200 nanoseconds, across crucial residues that play a role in stabilizing the RIPK2-XIAP complex. The dotted line at 0.35 nm indicates complete formation of H-bond.
