# Supplementary material for: AlphaFold-SFA: Accelerated sampling of cryptic pocket opening, protein-ligand binding and allostery by AlphaFold, slow feature analysis and metadynamics
Source: PLoS One. 2024 Aug 27;19(8):e0307226. doi: 10.1371/journal.pone.0307226 (PMC11349229; doi:10.1371/journal.pone.0307226)
Supplement: S1 Text — (PDF) [file pone.0307226.s024.pdf]

S1 Text: Choice of sequences for structure prediction:

The sequence of the protease domain of plasmepsin-II was used generated structural ensemble using ColabFold. The protease domain of the plasmepsin-II is a key drug target to develop novel antimalarial drugs.
Sequence:

LGSSNDNIELVDFQNIMFYGDAEVGDNQQPFTFILDTGSANLWVPSVKCTTAGCLTKHLYDSSKSRTYEKD
GTKVEMNYVSGTVSGFFSKDLVTVGNLSLPYKFIEVIDTNGFEPTYTASTFDGILGLGWKDLSIGSVDPIVVEL
KNQNKIENALFTFYLPVHDKHTGFLTIGGIEERFYEGPLTYEKLNHDLYWQITLDAHVGNIMLEKANCIVDSG
TSAITVPTDFLNKMLQNLDVIKVPFLPFYVTLCNNSKLPTFEFTSENGKYTLEPEYYLQHIEDVGPGLCMLNIIG
LDFPVPTFILGDPFMRKYFTVFDYDNHSVGIALAKKNL

The kinase domain of serine-threonine kinase, RIPK2 is critical to develop ATP competitive inhibitors as well plays crucial role in engaging with E3 ligase, XIAP. RIPK2 and XIAP interaction is pivotal in modulating downstream signaling pathways for the production of pro-inflammatory cytokines and immune responses.
Sequence:

AICSALPTIPYHKLADLRYLSRGASGTVSSARHADWRVQVAVKHLHIHTPLLDSERKDVLREAEILHKARFSYI
LPILGICNEPEFLGIVTEYMPNGSLNELLHRKTEYPDVAWPLRFRILHEIALGVNYLHNMTPPLLHHDLKTQNI
LLDNEFHVKIADFGLSKWRMMSLSQSRSSKSAPEGGTIIYMPPENYEPGQKSRASIKHDIYSYAVITWEVLSR
KQPFEDVTNPLQIMYSVSQGHRPVINEESLPYDIPHRARMISLIESGWAQNPDERPSFLKCLIELEPVLRTFEEI
TFLEAVIQLK
