# Supplementary material for: AlphaFold-SFA: Accelerated sampling of cryptic pocket opening, protein-ligand binding and allostery by AlphaFold, slow feature analysis and metadynamics
Source: PLoS One. 2024 Aug 27;19(8):e0307226. doi: 10.1371/journal.pone.0307226 (PMC11349229; doi:10.1371/journal.pone.0307226)
Supplement: Attachment — Submitted filename: response-reviewer.docx [file pone.0307226.s025.docx]

**Response to the Reviewers**

The authors appreciate fast review process and constructive comments by the PlosOne academic editor and the reviewers. We addressed each reviewer comments and added modifications/clarifications accordingly in the main text and the supplementary information (highlighted in BLUE). We thank the academic editor and the reviewers for their time and effort to enhance our manuscript.

**Reviewer #1:** 

1. Alphafold is a good structure prediction tool, but what is the advantage of using the conformations it generates as the starting conformations to generate CV compared to using a simple Steer MD and path cv? If it is just for taking rare conformations, some high-energy unreasonable conformations themselves may also have a bad effect on the prediction of CV. This part may require more explanation.

Answer: This is a great point. Bobrovs and coworkers previously used path CV in conjunction with traditional distance CVs in funnel metadynamics to account for protein dynamics (reference 41, paper: <https://pubs.acs.org/doi/10.1021/acs.jcim.2c00422> ). However, setting up a path-based CV is challenging as it needs 3D-co-ordinates of starting state, end state and intermediate states. Bobrovs and coworkers used existing crystal structures of plasmepsin II to set up a path CV which better explores flap dynamics necessary for ligand binding. However, SFA is a compressed representation of multiple CVs which does not need prior information of conformational states similar to path CVs. SFA trained on molecular dynamics simulations data captures underlying key features governing flap dynamics and cryptic pocket opening in plasmepsin II as highlighted in Figure 4 and 5. This makes SFA a robust alternative of path CV and allows more flexibility to incorporate within metadynamics protocol. We have added the following section in the manuscript:

“Previous study^41^ has highlighted the use of path CVs to incorporate protein structural dynamics, which accelerates the sampling of protein motions necessary for ligand binding. However, path CVs necessitate knowledge 3D structural co-ordinates of starting state, end state, and intermediate states along the transition pathway. Identifying these states is often challenging without prior structural information obtained from extensive experimental data or computational simulations. SFA trained on AlphaFold seeded molecular dynamics captures linear combinations of key structural features accounting for the conformational dynamics of protein without requiring *a* *priori* information.”

We would also like to highlight that steer MD is not a natural descriptor of complex biological motion highlighted in the manuscript which is a function of multiple degrees of freedom across different features. Steer MD would allow us to see ligand binding however it would fail to capture flap opening + Trp41, two key conformational events necessary to effectively sample multiple transitions of ligand unbinding and binding.

Authors raised a great question: Is it possible that incorporating rare high energy conformations has bad effect in prediction of CV? This is a great point, and the answer is YES. If we trained SFA directly on AlphaFold output, we would have incorporated random noise in our CV prediction. Molecular dynamics simulations initiated from AlphaFold structures not only ensure that the conformations are well-equilibrated but also guarantee that the training data for SFA represents an unbiased Boltzmann distribution.

We draw attention to Figures 9 (A, B), 10 and 12C, where the black dots represent AlphaFold-generated structures. It is evident that AlphaFold alone failed to sample active-to-inactive transitions. Had we trained SFA exclusively on AlphaFold output, we would have missed the key features necessary for capturing active-to-inactive transitions in apo RIPK2. The subsampling of AlphaFold structures introduces conformational diversity, which, when combined with molecular dynamics simulations, enables the sampling of essential structural motions. SFA then identifies these key structural motions as linear combinations.

The capacity to detect multidimensional structural features, often absent in AlphaFold output, allows SFA to serve as an effective CV within the metadynamics framework, thereby accelerating conformational sampling.

“SFA trained on molecular dynamics simulations seeded from AlphaFold structures captures Boltzmann-weighted structural features necessary to sample conformational transitions associated with cryptic pocket opening in plasmepsin II and allosteric dynamics in RIPK2. In the case of cryptic pocket opening, initiating multiple short unbiased molecular dynamics simulations from the diverse conformational ensemble generated by AlphaFold confers an advantage in conformational sampling, as this ensemble captures multiple degrees of freedom along key structural features (Figures 4 and 5).

Conversely, for RIPK2, AlphaFold alone failed to sample conformational transitions across key structural features involved in active-to-inactive transitions (Figures 9, 10, and 12). SFA trained solely on the AlphaFold-generated ensemble would have been unable to capture the critical transitions required for active-to-inactive transitions. However, short molecular dynamics simulations initiated from the AlphaFold-generated ensemble of RIPK2 which sampled structural heterogeneity of the activation loop (missing in the crystal structures of apo and holo RIPK2), captures Boltzmann-weighted structural features governing active-to-inactive transitions.

This highlights the importance of performing multiple short MD simulations starting from the AlphaFold-generated conformational ensemble, which not only equilibrates the structures but also samples Boltzmann-weighted transitions across key structural features involved in biologically significant conformational transitions.”

2. This method uses microsecond-level calculations when generating CV. If the corresponding computing resources are directly used to calculate metadynamics, can better convergence be achieved? What are the specific applications and advantages of this method?

Answer: It is well known that longer you perform metadynamics you will reach a asymptotic convergence limit (<https://pubs.aip.org/aip/jcp/article-abstract/143/23/234112/193998/A-perturbative-solution-to-metadynamics-ordinary?redirectedFrom=fulltext> ). To develop a fully connected Markov State Model (MSM) for estimating the free energy surface (FES) along key features, we would have required approximately 16 microseconds of unbiased molecular dynamics simulation data, as illustrated in **Figure S1 of the Supplementary Information**. In contrast, SFA-metadynamics enables FES prediction within a few hundred nanoseconds, underscoring its significant advantage over methods like MSM. **Moreover, MSM itself cannot be directly incorporated into enhanced sampling workflows.**

SFA offers the dual benefit of allowing the construction of an MSM based on a compressed representation of multidimensional features while also serving as a collective variable (CV) within enhanced sampling frameworks such as metadynamics. This versatility extends its applicability as a unifying approach, bridging MSM and enhanced sampling methodologies. We have added the following in the discussion to highlight that

“SFA can also act as a reduced space on which one can build MSM. Further, conformations which contribute to the first few slow features can be used as seeds to launch small MD simulations. These MD simulations can be stitched together using MSM to predict thermodynamics and kinetics associated with conformational transitions. Kinetics extracted from MSM built on top of SFA can be directly compared with infrequent metadynamics^51^ with slow features as CVs. In essence, SFA emerges as an innovative technique that bridges the gap in the estimation of kinetics linked to rare events, whether one uses MSM or metadynamics.”

In our forthcoming paper, we will demonstrate how SFA also serves as an effective tool for identifying key structural features to initiate adaptive sampling simulations, thereby uncovering conformational allostery upon ligand binding.

3. It is easy to see the acceleration of this method in sampling, but the convergence effect does not seem to be reflected. The results in the article are all variables with specific physical meanings, such as in Figure 9 (B, D). Is this the result of reweighting after the selected CV converges?

Answer: The FES was projected along different features after reweighting. This is highlighted in the main text: “Unbiased free energy surfaces along different features were extracted using the reweighting protocol developed by *Tiwary and Parrinello*^37^.”

The convergence along CVs for plasmepsin II and RIPK2 has been described in detail in the Supplementary Information. We have highlighted this in the main text as follows: “Comparison of reweighted free energy surfaces at different time intervals from metadynamics simulations highlighted the convergence (**Figure S6 in Supporting Information**) for the choice of force field and water model.”

Accelerated sampling along first two slow features (**Figure S5 in Supporting Information**) , reweighted free energy surface along Trp41 𝜒1 and 𝜒2 along different time intervals (**Figure S6 in Supporting Information**) and projection of the metadynamics reweighting factor (see more here: <https://pubs.acs.org/doi/10.1021/jp504920s> ) as a function of simulation time clearly highlights the relative convergence of the metadynamics simulations within the simulation time. Similar projections have been highlighted for RIPK2 systems in **Figure S12 and S13 of the Supplementary Information.**

**Reviewer 2:**

1.The paper should introduce the biological background of cryptic pockets to enhance understanding for general readers.

Answer: We have introduced the background of cryptic pocket and allostery in the main text. Highlighted below:

“Cryptic pockets have emerged as a frontier in modern drug discovery, offering new opportunities to target proteins previously considered "*undruggable*." These hidden binding sites are not apparent in the protein's apo state but become accessible upon interaction with small molecules or during *rare* conformational transitions. Sampling cryptic pocket opening in apo proteins is of significant interest in drug discovery, as targeting these pockets has the potential to yield highly selective and potent inhibitors with unique structural features distinct from catalytic sites or known binding interfaces. The strategy of targeting cryptic pockets has proven attractive in developing selective inhibitors against challenging targets, such as plasmepsin-II, a key enzyme in antimalarial drug discovery. Similar to cryptic pockets, allostery in protein kinases is a rare conformational event which represents a complex signaling pathway via which changes in the active site of kinase induces conformational changes at a distal site. Understanding allostery in kinases is key to uncovering the mechanisms by which these enzymes interact with protein partners and regulate downstream signaling across several disease pathways. Elucidating allosteric fingerprints is key to gain insights into kinase function beyond catalytic activity, potentially revealing new strategies for therapeutic intervention in various pathological conditions.”

2. Clarification is needed on which sequences were selected to generate the structural ensemble using the ColabFold implementation of AlphaFold. The rationale for selecting these sequences and their biological relevance should be provided.

Answer: We have added a new section titled “Choice of sequences for structure prediction:” in the Supplementary Information. We have added the following in the main text:

“The specific sequences used to generate the conformational ensemble are detailed in the 'Choice of sequences for structure prediction' section of the Supporting Information.”

3. Several figures lack clarity, hindering the assessment of certain conclusions:
In Figure 10, Arg65 forms an H-bond interaction with Ser168, but Ser168 is not labeled.

Answer: We have introduced a new **Figure 11 (previous numbering Figure 10) highlighting the Arg65 and Ser168 H-bond and relative orientation of the key residues.**

In Figure 8, the key residues are not clearly labeled.

We have added the label for Phe165 in the updated Figure 9 (previously Figure 8).

In Figure 11 (D), the location of the salt-bridge interaction is not clearly indicated.

Highlighted as a double ended arrow in the modified Figure 12 (D) (previously Figure 11)

**Reviewer 3:**

1. While utilizing AI or AlphaFold to predict protein structures is a research trending area, but it is not closely related to the main thrust of this paper and does not play a significant role in it. AlphaFold (AF) performs structural predictions based on experimental data and known information, but the structures within the ensemble it produces may not have physical meaning. What is the significance of using these structures as initial structures for further MD simulations? Alternatively, the author should compare the differences and advantages, if any, between computational simulations using a single AF predicted structure versus an ensemble of structures as the initial structure. Otherwise, it is completely unnecessary.

Answer: To test reviewer’s hypothesis, we generated conformation from the AlphaFold3 web server (<https://alphafoldserver.com>) and performed combined 3 microsecond of unbiased molecular dynamics simulation and projected the distribution along Trp41 𝜒1 and 𝜒2 angles. It is clear that starting unbiased molecular dynamics simulations from closed conformation fails to capture open < -- > close transition as highlighted in Figure 4 (A, C) of the manuscript.

We will like to highlight the reviewer two of the key papers in this field which describe in the detail the sampling benefit using the stochastic subsampling of AlphaFold’s input MSA: Paper 1: <https://pubs.acs.org/doi/epdf/10.1021/acs.jctc.2c01189> and

Paper 2: <https://pubs.acs.org/doi/abs/10.1021/acs.jctc.3c00290>

Figure. A: Conformations generated by AlphaFold 3 webserver (<https://alphafoldserver.com> ), B: Trp41 adapts closed conformation in the conformations generated by AlphaFold 3 webserver. C: Projection of unbiased free energy surface along Trp41 𝜒1 and 𝜒2 angles highlight lack of sampling of cryptic pocket open states (PDB: 2BJU, 4Z22).

We have also added the following section in the revised manuscript

“SFA trained on molecular dynamics simulations seeded from AlphaFold structures captures Boltzmann-weighted structural features necessary to sample conformational transitions associated with cryptic pocket opening in plasmepsin II and allosteric dynamics in RIPK2. In the case of cryptic pocket opening, initiating multiple short unbiased molecular dynamics simulations from the diverse conformational ensemble generated by AlphaFold confers an advantage in conformational sampling, as this ensemble captures multiple degrees of freedom along key structural features (Figures 4 and 5).

Conversely, for RIPK2, AlphaFold alone failed to sample conformational transitions across key structural features involved in active-to-inactive transitions (Figures 9, 10, and 12). SFA trained solely on the AlphaFold-generated ensemble would have been unable to capture the critical transitions required for active-to-inactive transitions. However, short molecular dynamics simulations initiated from the AlphaFold-generated ensemble of RIPK2 which sampled structural heterogeneity of the activation loop (missing in the crystal structures of apo and holo RIPK2), captures Boltzmann-weighted structural features governing active-to-inactive transitions.

This highlights the importance of performing multiple short MD simulations starting from the AlphaFold-generated conformational ensemble, which not only equilibrates the structures but also samples Boltzmann-weighted transitions across key structural features involved in biologically significant conformational transitions.”

2. This paper applies the method of Slow Feature Analysis (SFA) to analyze the results of unbiased MD simulations and uses the so-called "slow features" obtained as reaction coordinates for metadynamics. Let's set aside whether this method can truly capture slow features or not. The reduced-dimensional feature values obtained from SFA do not inherently possess physical meanings. The author needs to provide further details on how to add a Gaussian potential to this Collective Variable (CV) in metadynamics and conduct subsequent sampling.

**Answer**: Slow feature analysis represents linear combination of sin/cos transformed 𝜒1 and 𝜒2 angles for chosen residue selection. As an example, the physical meaning of residues captured by SFA is highlighted in **Figure S3 of Supporting Information**. It is clear the SFA manages to capture 𝜒1 and 𝜒2 angles corresponding Trp41 and Tyr77 as key motions governing cryptic pocket opening in plasmepsin II. Our manuscript overall described how these motions are connected to crypto pocket opening. This highlights that SFA is capable of capturing physically relevant conformational degrees of freedom.

The metadynamics protocol is described in the “metadynamics” section. Plumed files area accessible in the GitHub repo: <https://github.com/sbhakat/AlphaFold-SFA/tree/main>

3. In the Funnel metadynamics based on SFA features, the author did not set a reaction coordinate for the distance between the ligand and the protein. How can we ensure that rapid sampling can be achieved, and the ligand-protein binding process can be derived?

**Answer**: “Traditional funnel metadynamics was performed using the distance between the center of mass (COM) of ligand and Cγ atoms of catalytic Asp dyads (Asp 34 and 214) as CV.”

Highlighted in BLUE in the main text. It is critical to note this CV does not capture the dynamics of protein that is flap opening and cryptic pocket opening two key factors governing rebinding of the ligand.

4. In the Funnel Metadynamics (FM) based on SFA features, the SFA features are obtained by learning the system without ligands. How can we ensure that the changes in the protein and the reaction coordinates after introducing ligands are not affected by the ligands?

**Answer**: The ligand-protein complex itself does not do any conformational change hence it is impossible to capture the protein conformational changes necessary for ligand rebinding if one only performs unbiased MD simulation of the holo complex.

SFA captures two key motions necessary for rebinding of the ligand to the deep cryptic pocket of plasmepsin-II: a) flap opening and b) cryptic pocket opening described by Trp41 𝜒1 and 𝜒2 angles. Hence SFA in combination with the distance CV (distance between the center of mass (COM) of ligand and Cγ atoms of catalytic Asp dyads (Asp 34 and 214) as CV) not only accelerates ligand unbinding but allows conformational changes in protein which is necessary for ligand rebinding (**Figure 6 and 7**).

5. What is the connection between the features learned by the author through SFA and the reaction coordinates used to construct the free energy surface in Figure 4/5? Why not create a free energy surface projected onto the SFA features?

**Answer**: SFA captures key conformational transitions associated with deep cryptic pocket opening in plasmepsin II as highlighted in Figure S3 in Supporting Information. Reweighted free energy surfaces along the dihedral’s angles highlighted the accelerated sampling by SFA-metadynamics compared to multi microsecond of unbiased molecular dynamics simulations.

Thanks for suggestion. We have also projected FES along SF1 and SF2, see Figure S4 in the Supporting Information.
